# Effectiveness of Peri-Discharge Complex Interventions for Reducing 30-Day Readmissions among COPD Patients: Overview of Systematic Reviews and Network Meta-Analysis

**DOI:** 10.5334/ijic.6018

**Published:** 2022-02-03

**Authors:** Claire C. W. Zhong, Charlene H. L. Wong, William K. W. Cheung, Eng-kiong Yeoh, Chi Tim Hung, Benjamin H. K. Yip, Eliza L. Y. Wong, Samuel Y. S. Wong, Vincent C. H. Chung

**Affiliations:** 1 Jockey Club School of Public Health and Primary Care, The Chinese University of Hong Kong, Shatin, HK; 2 School of Chinese Medicine, The Chinese University of Hong Kong, Shatin, HK

**Keywords:** 30-day readmissions, chronic obstructive pulmonary disease, peri-discharge complex Interventions, overview of systematic reviews, network meta-analysis

## Abstract

**Background:**

An overview of systematic reviews(SRs) and network meta-analysis(NMA) were conducted to evaluate the comparative effectiveness of peri-discharge complex interventions for reducing 30-day readmissions among chronic obstructive pulmonary disease(COPD) patients.

**Methods:**

Five databases were searched for SRs of randomized controlled trials(RCTs). An additional search was conducted for updated RCTs from database inception until Jun 2020. Pooled effect of peri-discharge complex interventions was assessed using random-effect pairwise meta-analyses. Comparative effectiveness across different peri-discharge complex interventions was evaluated using NMA.

**Results:**

Nine SRs and 11 eligible RCTs(n = 1,422) assessing eight different peri-discharge complex interventions were included. For reducing 30-day all-cause readmissions, pairwise meta-analysis showed no significant difference between peri-discharge complex interventions and usual care, while NMA indicated no significant differences among different peri-discharge complex interventions as well as usual care. For reducing 30-day COPD-related readmissions, peri-discharge complex interventions were significantly more effective than usual care (pooled RR = 0.45, 95% CI:0.24–0.84).

**Conclusions:**

Peri-discharge complex interventions may not differ from usual care in reducing 30-day all-cause readmissions among COPD patients but some are more effective for lowering 30-day COPD-related readmission. Thus, complex intervention comprising core components of patient education, self-management, patient-centred discharge instructions, and telephone follow up may be considered for implementation, but further evaluation is warranted.

## Introduction

Chronic obstructive pulmonary disease (COPD) is a progressive and life-limiting disease that imposes a huge clinical and economic burden on health system [1]. In 2019, the number of COPD patients was approximately 212 million worldwide [2]. The World Health Organization predicts that by 2030, it will be the third leading cause of mortality globally [3]. COPD is also one of the most common causes for 30-day readmissions [4,5], which are considered as a highly common, expensive, and unfavourable outcome of health systems [6,7,8,9]. In 2011, the 30-day readmission rates among COPD patients in the United States and Hong Kong were 22% [10] and 24% [11], respectively. The 30-day readmissions were associated with lower quality of life [12,13] and irreversible damage on patients exercise capacity, muscle strength, and physical activity level. It also increased mortality [14] and health care expenditure [15] for COPD patients.

Fortunately, it is estimated that up to 37% of 30-day readmissions among COPD patients are preventable [16]. To respond to the call for reducing 30-day readmissions in different health systems such as the United States [17] and the UK [18], numerous peri-discharge complex interventions have been proposed, evaluated, and implemented [19,20]. A previous systematic review (SR) indicated that discharge support intervention for COPD patients was significantly more effective in reducing 30-day all-cause readmissions when compared with usual care [21]. According to another meta-analysis of eight randomized controlled trials (RCTs), discharge support intervention was more effective in reducing 6-month all-cause readmissions than usual care among COPD patients [22]. Besides, findings of an SR showed that pulmonary rehabilitation was more effective than usual care in reducing COPD-related readmissions within 3–12 months after discharge [23].

The key goal of these peri-discharge complex interventions is to ensure a seamless transition from inpatient to outpatient care. Quality of such transition is considered as one of the key factors associated with successful prevention of avoidable 30-day hospital readmissions [24]. These peri-discharge complex interventions can be considered as a form of integrated care, as it fits into at least two common definitions of integrated care. Firstly, from a healthcare manager’s perspective, delivery of peri-discharge complex interventions demands the creation and maintenance of a coordinated, interdependent service structure between individual providers and organizations for driving the common goal of reducing avoidable readmissions [25]. Secondly, from a chronic care perspective, these peri-discharge complex interventions seek to manage multiple needs of COPD patients by linking services from different providers along the continuum of care [26].

In practice, the nature and components of peri-discharge complex interventions vary across health systems. For example, discharge support intervention can be included in both pre-discharge preparation and post-discharge care for COPD patients [21]. For pre-discharge preparation, discharge rounds and discharge protocols implemented by multidisciplinary teams would be tailored in accordance with their multiple needs. For post-discharge care, patient empowerment and telephone follow-up would be provided to encourage patients’ active participation in self-care. In addition, efforts could be made to improve the communication between hospital-based specialists, primary care physicians and social care professionals to enhance intersectional collaboration and continuity of care. These initiatives would be provided to COPD patients as a bundle with the aim to prevent avoidable hospital readmissions.

Existing studies have summarized the effectiveness of various peri-discharge complex interventions for reducing readmissions at different time points [21,22,23,27,28], but their comparative effectiveness is uncertain. In this overview of SRs and network meta-analysis (NMA), we aimed to evaluate the comparative effectiveness of different peri-discharge complex interventions on reducing 30-day readmissions among COPD patients.

## Research Methods

This study was reported according to the PRISMA extension statement for NMA [29]. Protocol was registered in the PROSPERO database (Registration No. CRD42020204719). Detailed inclusion criteria of SRs and RCTs are shown in ***Table 1***.

**Table 1:** Inclusion criteria for eligible systematic reviews (SRs) and randomized controlled trials (RCTs).


INCLUSION CRITERIA	ELIGIBLE SRS	ELIGIBLE RCTS

Participants	Adult patients (≥18 years) admitted from the community to a hospital inpatient ward for 24 hours or more; and The diagnosis of the initial admission was COPD. Participants with behavioural health issues, paediatric, or obstetric admission were excluded.	

Interventions	Any pre-emptive peri-discharge complex interventions for reducing readmissions.	

Comparisons	Any types of control as comparisons, including usual care.	

Outcomes	Eligible SRs should report readmission outcomes in both intervention and control groups.	Eligible RCTs should report 30-day all-cause or 30-day COPD-related readmissions in both intervention and control groups


### Inclusion criteria for SRs

SR is defined as an “endeavour to identify, appraise, and synthesize all the evidence that fulfils pre-specified eligibility criteria to answer a specific research question” in the Cochrane Handbook version 6 [30]. Accordingly, SRs eligible for this overview should fulfil all of the following characteristics [31]: i) state clear research questions; ii) describe a reproducible search strategy including databases, search platforms/engines, search date, and complete search strategy; iii) report inclusion and exclusion criteria; iv) include screening methods; v) critically appraise risk of bias of included studies; vi) report data analysis methods which allow reproducibility, vii) being published in English or Chinese; and viii) satisfy the following criteria for participants, interventions, comparisons, and outcomes:

#### Participants

Participants should be adult patients (≥18 years) admitted from the community to a hospital inpatient ward for 24 hours or more with a diagnosis of COPD. Participants with behavioural health issues, pediatric, or obstetric admission were excluded.

#### Interventions and comparisons

Interventions should be any pre-emptive peri-discharge complex interventions for reducing readmissions, which were compared with any types of control as comparisons, including usual care. In this study, peri-discharge complex interventions referred to interventions comprising multiple interacting components delivered during the peri-discharge process [32]. Usual care was defined as routine care provided by hospitals, as prompted by the needs of the patients. There was no restriction on the number of components included in both peri-discharge complex interventions and in control interventions. Aside from hospitals, peri-discharge complex interventions implemented in the following settings were also eligible: convalescent hospitals, nursing homes, hospices, primary care, the community, or patients’ homes.

#### Outcomes

Eligible SRs should report readmission outcomes in both intervention and control groups among the embedded RCTs. Details are listed in the paragraph below.

#### Inclusion criteria of RCTs embedded in SRs

After including all eligible SRs, embedded RCTs were extracted and assessed for eligibility criteria as well. To be included, an embedded RCT should fulfill the same criteria for participants, interventions, and comparisons for SRs as abovementioned. For outcomes, eligible RCTs should report 30-day all-cause or 30-day COPD-related readmissions in both intervention and control groups. Primary and main secondary outcomes are 30-day all-cause and 30-day COPD-related readmissions, respectively. These two outcomes are chosen because they are considered to be modifiable by appropriate peri-discharge complex interventions among policy makers [33]. For instance, the US Hospital Readmission Reduction Program (HRRP) regarded all unplanned readmissions within 30 days after discharge as an indicator of poor performance, which would lead to financial penalties to hospitals if the risk-standardized 30-day readmission rates are higher than expected [34,35].

Other secondary outcomes included 3-month and 6-month all-cause readmissions, as well as 30-day mortality. The 3-month and 6-month readmission outcomes are selected as they can reflect the medium-term impact of peri-discharge complex interventions. These readmissions are known to be associated with a substantial risk of mortality and adverse impacts on health-related quality of life [36,37]. Lowering readmission rate may inadvertently increase mortality rate, therefore this is considered as a secondary outcome of interest as well [35].

### Literature search

We searched for SRs in MEDLINE, EMBASE, Cochrane Database of Systematic Reviews, Global Health, and AMED from the databases’ inception till August 2019. We applied specialized filters with balanced sensitivity and specificity for SRs in MEDLINE and EMBASE. No restrictions on publication status were imposed.

To be more comprehensive, we conducted an updated search for potentially eligible RCTs in MEDLINE, EMBASE, and Cochrane Central Register of Controlled Trials published from databases’ inception till Jun 2020. Detailed search strategies for SRs and RCTs are shown in Appendix 1a-1b, respectively. Their eligibility criteria were the same as illustrated in ***Table 1***.

### Literature selection, data extraction, methodological quality assessments, risk of bias assessment, and quality of evidence rating

Literature selection, data extraction, methodological quality assessments [38], risk of bias assessment [39], and quality of evidence rating [40,41] were conducted by two reviewers (CW, CZ) independently. Disagreements were resolved by discussion. A third reviewer (VC) was consulted to settle unsolved discrepancies. Details of literature selection and data extraction could be found in Appendix 2.

We used the validated AMSTAR 2 instrument [38] to appraise methodological quality of included SRs. Overall methodological quality of each SR was appraised as high, moderate, low, or critically low. We applied the Cochrane Risk of Bias Tool 2 [39] to assess risk of bias of included RCTs. Overall risk of bias of each RCT was judged as low risk of bias, some concerns, or high risk of bias based on the answers to the signaling questions across the following five domains, including i) bias arising from the randomization process, ii) bias due to deviations from intended interventions, iii) bias due to missing outcome data, iv) bias in measurement of the outcome, and v) bias in selection of the reported result [39]. We adopted the Grading of Recommendations Assessment, Development, and Evaluation (GRADE) approach to assess the overall quality of evidence, respectively for pairwise meta-analyses [40] and NMA [41]. The quality of evidence for each outcome was graded as high, moderate, low, and very low [40–41].

In each included RCTs, peri-discharge complex interventions for reducing readmissions consisted of different components. To facilitate analysis, we coded components of different peri-discharge complex interventions based on a published classification framework [42] (see Appendix 3). Two reviewers (CW, CZ) performed the coding process independently after co-piloting, and reached a consensus on an unitified coding result after discussion. A third reviewer (VC) would make the decision if consensus cannot be reached for individual interventions.

### Data analysis

#### Pairwise meta-analyses

Following standard methodology in the field [43], we first conducted pairwise meta-analyses and then NMA for data analysis. We conducted pairwise random-effect meta-analyses of comparing peri-discharge complex interventions with controls using Revman 5.3. We used pooled risk ratios (RRs) with 95% confidence intervals (CIs) to present dichotomous data. We used I^2^ values to indicate the level of heterogeneity, with I^2^ <25% as low level, 25–50% as moderate level, and >50% as high level [44].

We conducted a sensitivity analysis by only pooling RCTs with an overall low risk of bias on the primary outcome of 30-day all-cause readmissions. We also conducted a subgroup analysis on the primary outcome by stratifying RCTs based on different types of control interventions.

#### Network meta-analysis

NMA is a group of methods for visualizing and analyzing a wider picture of existing evidence, which allows assessment of comparative effectiveness among different interventions [45]. It generates indirect evidence (estimates between different interventions via common comparator) when direct evidence (head-to-head estimates of different interventions) is unavailable [46]. In this overview of SRs, the common peri-discharge complex intervention was served as a bridge to conduct NMA, so we could explore, relatively speaking, the most effective intervention package for the primary and secondary outcomes among all included interventions [47].

NMA was conducted using STATA version 14.0 [45]. Comparative effectiveness results of all possible pairs of comparisons were summarized with odds ratios (ORs) and associated 95% CIs [48] The surface under the cumulative ranking curve (SUCRA) was used to provide an effectiveness hierarchy ranking [49]. The probability that an intervention being the most effective option, the second-best option, and so on was deduced, comparatively [49]. The larger the SUCRA, the higher effectiveness ranking the intervention would have.

Consistency of direct and indirect evidence on the same comparison is a key assumption of NMA [49,50]. The amount of inconsistency was measured by the inconsistency factor, which refers to the absolute mean difference between direct and indirect comparisons within a loop [51]. We used the separating indirect from direct evidence (SIDE) approach to calculate inconsistency factors, associated p values and 95% CIs [51]. When the p-values of inconsistency factors are smaller than 0.05, statistically significant inconsistency is detected [51]. In this case, quality of evidence would be rated down one or two levels for serious or very serious inconsistency, respectively in accordance to the GRADE methodology [41].

Optimal interpretation of NMA results requires considerations on the effect estimates as well as quality of evidence beyond ranking. To ensure appropriate interpretation, we applied an established minimally contextualized framework to facilitate simultaneous consideration of these aspects [52]. In this framework, effectiveness of peri-discharge complex interventions was categorized based on the network estimates, their associated quality of evidence, and SUCRA results. As a first step, we classified these interventions into two groups based on network estimates as follows:

1. Group 1: interventions which are not different from usual care.
2. Group 2: interventions which are superior to at least one intervention in Group 1.

Secondly, in each group, we further divided these interventions into two categories based on certainty of evidence: i) high certainty category containing interventions supported by moderate or high quality of evidence; and ii) low certainty category containing interventions supported by low or very low quality of evidence. Finally, we checked consistency between the network estimates among all possible pairs of comparisons and SUCRA rankings, so as to finalize the classification of all interventions.

## Results

### Results on literature search and selection

**Figure 1:**
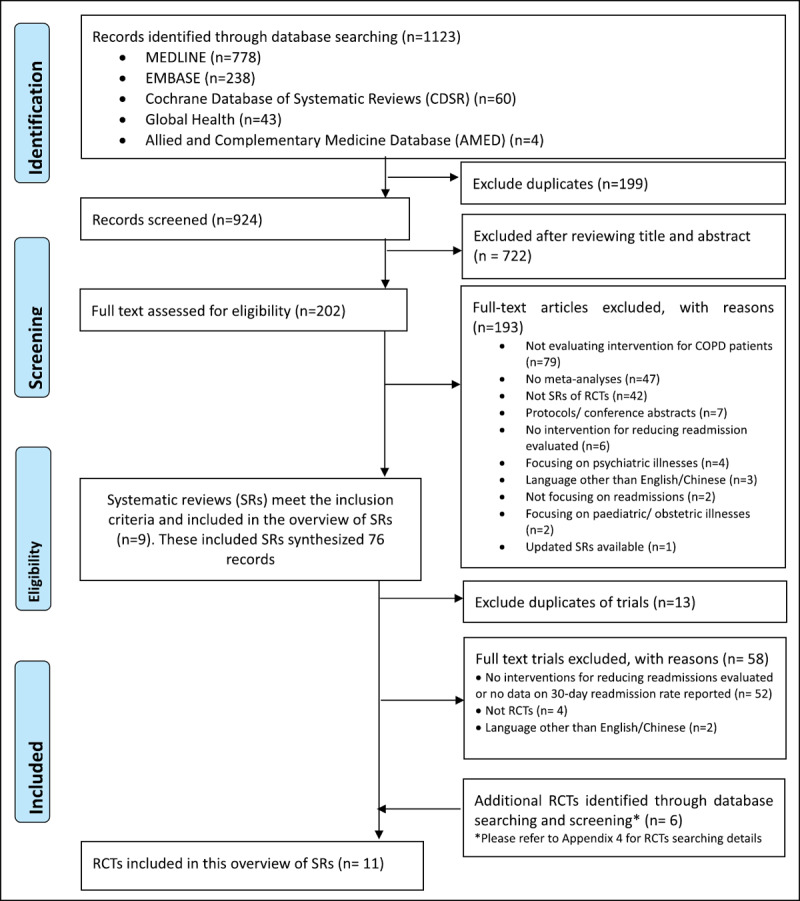
Flowchart of literature search and selection for systematic reviews and randomized controlled trials. Keys: SRs: systematic reviews; RCTs: randomized controlled trials.

A total of nine SRs were identified and considered to be eligible (Appendix 5a). These nine SRs synthesized 76 primary studies, of which 71 were excluded due to the following: being duplicates (n = 13); no intervention for reducing readmission evaluated, or no data on 30-day readmission rate reported (n = 52); not RCTs (n = 4); written in languages other than English/Chinese (n = 2). The additional literature search identified six RCTs that were considered eligible (Appendix 4). Therefore, a total of 11 RCTs were included (Appendix 5b). Details of the literature search and selection process are presented in ***Figure 1***.

### Characteristics of included RCTs

#### Participants

Characteristics of the included RCTs are presented in ***Table 2***. They included a total of 1,422 COPD patients, with sample sizes varying from 30 to 253. The mean age range was 64.4 to 75.3 years.

**Table 2:** Main characteristics of included randomized controlled trials (RCTs) (n = 11).


FIRST AUTHOR, YEAR OF PUBLICATION	COUNTRY	FOLLOW-UP PERIOD OF THE STUDY	INTERVENTIONS/COMPARATORS	NO. OF PATIENTS IN THE GROUP (A/R)	AGE RANGE/MEAN ± SD (YEARS)	GENDER N (% MALE)	PRIMARY OUTCOME: 30-DAY ALL-CAUSE READMISSIONS: NO. OF EVENTS (%)	SECONDARY OUTCOME: 30-DAY COPD-RELATED READMISSIONS: NO. OF EVENTS (%)	SECONDARY OUTCOME: 30-DAY MORTALITY: NO. OF EVENTS (%)	SECONDARY OUTCOME: 3-MONTH ALL-CAUSE READMISSIONS: NO. OF EVENTS (%)	SECONDARY OUTCOME: 6-MONTH ALL-CAUSE READMISSIONS: NO. OF EVENTS (%)

Benzo 2016	USA	Sept 2010–Aug 2014	Discharge rehabilitation	108/108	67.9 ± 9.8	46(42.6)	5(4.6)	2(1.9)	NR	15(13.9)	28(25.9)

			Usual care	107/107	68.1 ± 9.2	51(47.7)	12(11.2)	10(9.4)	NR	27(25.2)	40(37.4)

Cotton 2000	UK	NR	Early discharge intervention	41/41	65.7 ± 1.6	19(46.3)	6(14.6)	NR	NR	NR	NR

			Usual care	40/49	68.0 ± 1.2	16(32.7)	6(12.2)	NR	NR	NR	NR

Eaton 2009	New Zealand	Jun 2005–Oct 2006	Discharge rehabilitation	47/47	70.1 ± 10.3	21(44.7)	NR	3(6.4)	NR	11(23.4)	NR

			Discharge education	50/50	69.7 ± 9.4	21(42.0)	NR	4(8.0)	NR	16(32.0)	NR

Hornikx 2015	Belgium	Apr 2013–Apr 2014	Home based telemedicine	12/15	66 ± 7	8 (53.3)	4(26.7)	NR	NR	NR	NR

			Rehabilitation education	15/15	68 ± 6	9 (60.0)	6(40.0)	NR	NR	NR	NR

Jabkobsen 2015	Denmark	Jun 2010– Dec 2011	Home based telemedicine	29/29	NR	11 (37.9)	8(27.6)	NR	NR	10(34.5)	13(44.8)

			Usual care	28/28	NR	11 (39.3)	6(21.4)	NR	NR	11(39.3)	14(50.0)

Jennings 2014	USA	Feb 2010–Apr 2013	Discharge coordinator intervention	93/93	64.9 ± 10.9	40(43.4)	18(19.4)	NR	NR	NR	NR

			Discharge education	79/79	64.4 ± 10.5	37(46.8)	18(22.8)	NR	NR	NR	NR

Johnson 2016	UK	Jan 2013–Sep 2014	Supported self-management program	35/39	67.6 ± 8.5	15(38.5)	NR	5(12.8)	NR	12(30.8)	NR

			Follow up appointment	36/39	68.3 ± 7.7	13(33.3)	NR	10(25.6)	NR	13(33.3)	NR

Kwok 2004	Hong Kong	Mar 1999– Aug 2000	Supported self-management program	70/77	75.3 ± 7.0	56(72.7)	33(42.9)	NR	NR	NR	53(68.8)

			Follow up appointment	79/80	74.2 ± 5.7	55(68.8)	29(36.3)	NR	NR	NR	49(61.3)

Lainscak 2013	Slovenia	Nov 2009–Dec 2011	Discharge coordinator intervention	118/118	71 ± 9	81(68.6)	7(5.9)	3(2.5)	1(0.8)	25(21.2）	37(31.4)

			Rehabilitation education	135/135	71 ± 9	101(74.8)	8(5.9)	8(5.9)	6(4.4)	39(28.9)	60(44.4)

Lavesen 2016	Denmark	Dec 2010–May 2012	Early discharge intervention	101/119	69.7 ± 10.3	46 (38.7)	25(21)	NR	2(1.7)	NR	NR

			Usual care	77/94	70.9 ± 9.8	37 (39.4)	22(23.4)	NR	3(3.2)	NR	NR

Wong 2005	Hong Kong	NR	Supported self-management program	30/30	72.8 ± 8.3	27(90.0)	5(16.7)	NR	NR	NR	NR

			Usual care	30/30	74.4 ± 7.4	20(66.7)	8(26.7)	NR	NR	NR	NR


*Notes*: A: number of patients analysed; R: number of patients randomized; SD: standard deviation; NR: not reported. COPD: Chronic Obstructive Pulmonary Disease. Usual care is defined as routine care provided by the hospital.

#### Interventions

There were five peri-discharge complex interventions evaluated in the intervention group: discharge coordinator intervention (n = 2), discharge rehabilitation (n = 2), early discharge intervention (n = 2), home based telemedicine (n = 2), and supported self-management program (n = 3). Components of each peri-discharge complex intervention and their definitions are presented in ***Table 3*** and Appendix 3.

**Table 3:** Components of peri-discharge complex interventions evaluated in included randomized controlled trials (RCTs).


PERI-DISCHARGE COMPLEX INTERVENTIONS	RCTS	COMMON COMPONENTS	CA	CM	DP	FS	PC	PE	PI	RI	SM	TE	TM

Discharge coordinator intervention	Jennings 2014	CA+PE+PI+TE	1	0	0	0	0	1	1	0	0	1	0

	Lainscak 2013	CA+PE+PI+TE	1	0	0	0	0	1	1	0	0	1	0

Discharge education^a^	Eaton 2009	PE+SM	0	0	0	0	0	1	0	0	1	0	0

	Jennings 2014	PE+SM	0	0	0	0	0	1	0	0	1	0	0

Discharge rehabilitation	Benzo 2016	DP+PC+RI+SM	0	0	1	0	1	0	0	1	1	0	0

	Eaton 2009	DP+PC+RI+SM	0	0	1	0	1	0	0	1	1	0	0

Early discharge intervention	Cotton 2000	CM+DP+TE	0	1	1	0	0	0	0	0	0	1	0

	Lavesen 2016	CM+DP+TE	0	1	1	0	0	0	0	0	0	1	0

Follow up appointment^b^	Johnson 2016	FS+PC	0	0	0	1	1	0	0	0	0	0	0

	Kwok 2004	FS+PC	0	0	0	1	1	0	0	0	0	0	0

Home based telemedicine	Hornikx 2015	SM+TM	0	0	0	0	0	0	0	0	1	0	1

	Jabkobsen 2015	SM+TM	0	0	0	0	0	0	0	0	1	0	1

Rehabilitation education^c^	Hornikx 2015	PE+RI	0	0	0	0	0	1	0	1	0	0	0

	Lainscak 2013	PE+RI	0	0	0	0	0	1	0	1	0	0	0

Supported self-management program	Johnson 2016	PE+PI+SM+TE	0	0	0	0	0	1	1	0	1	1	0

	Kwok 2004	PE+PI+SM+TE	0	0	0	0	0	1	1	0	1	1	0

	Wong 2005	PE+PI+SM+TE	0	0	0	0	0	1	1	0	1	1	0


*Notes*: CA: Case Management; CM: Timely Primary Care Provider Communication; DP: Discharge planning; FS: Follow-Up Scheduled; PC: Provider Continuity; PE: Patient Education; PI: Patient Centred Discharge Instructions; RI: Rehab Intervention; SM: Self-Management; TE: Telephone follow up; TM: Telemonitoring. *: Value of “0” means that the component (column) was not presented in the complex intervention package. †: Value of “1” means that the component (column) was presented in the complex intervention package. a: Discharge education is the control intervention of Eaton 2009 and Jennings 2014. b: Follow up appointment is the control intervention of Johnson 2016 and Kwok 2004. c: Rehabilitation education is the control intervention of Hornikx 2015 and Lainscak 2013. Definition for each component could be found in Appendix 3.

#### Controls

Different peri-discharge complex interventions were evaluated as controls among the included studies: discharge education (n = 2), follow up appointment (n = 2), rehabilitation education (n = 2). Components of peri-discharge complex interventions serving as controls are presented in ***Table 3***. The remaining five studies reported the use of usual care as control, which was defined as routine care provided by the hospital. Detailed contents of usual care were not mentioned in these five studies.

#### Methodological quality of included SRs and risk of bias among included RCTs

Amongst the nine included SRs, methodological quality of six SRs was moderate (66.7%). Two (22.2%) were appraised as low, and one (11.1%) as critically low (Appendix 6). For the 11 included RCTs, we judged the overall risk of bias of four (36.4%) RCTs as low, one (9.1%) as high, and the remaining six (54.5%) as having some concerns (Appendix 7a). Detailed results of the risk of bias assessment on each domain are presented in Appendix 7b.

### Results of pairwise meta-analyses

For the reduction of 30-day all-cause readmissions, there was no significant difference between peri-discharge complex interventions and controls from pairwise meta-analyses (pooled RR = 0.95, 95% CI: 0.76–1.19, I^2^ = 0%, 9 RCTs) (Appendix 8a). The overall quality of evidence was high (***Table 4***).

**Table 4:** Effect estimates and quality of evidence ratings for comparisons of pier-discharge complex interventions in pairwise meta-analyses sensitivity, and subgroup analysis.


OUTCOMES	STUDY DESIGN/PARTICIPANTS	RISK OF BIAS	INCONSISTENCY	INDIRECTNESS	IMPRECISION	PUBLICATION BIAS	POOLED RR (95% CI)	QUALITY

30-day all-cause readmissions	Nine RCTs/1247 participants	No serious^a^	No serious inconsistency	No serious indirectness	No serious imprecision	N/A	0.95(0.76,1.19)	⨁⨁⨁⨁ High

30-day COPD-related readmissions	Four RCTs/643 participants	No serious	No serious inconsistency	No serious indirectness	No serious	N/A	0.45(0.24,0.84)	⨁⨁⨁⨁ High

30-day mortality	Two RCTs/466 participants	No serious	No serious inconsistency	No serious indirectness	Serious imprecision^b^	N/A	0.35(0.09,1.34)	⨁⨁⨁◯ Moderate

3-month all-cause readmissions	Five RCTs/700 participants	No serious	No serious inconsistency	No serious indirectness	No serious	N/A	0.74(0.57,0.95)	⨁⨁⨁⨁ High

6-month all-cause readmissions	Four RCTs/682 participants	No serious	Serious inconsistency^c^	No serious indirectness	No serious	N/A	0.85(0.64,1.14)	⨁⨁⨁◯ Moderate

30-day all-cause readmissions (Sensitivity analysis focusing on RCTs with low risk of bias)	Three RCTs/444 participants	No serious	No serious inconsistency	No serious indirectness	Serious imprecision^b^	N/A	0.80(0.47,1.38)	⨁⨁⨁◯ Moderate

30-day all-cause readmissions (in subgroup 1: rehabilitation education as control interventions)	Two RCTs/283 participants	No serious	No serious inconsistency^d^	No serious indirectness	Very serious imprecision^e^	N/A	0.83(0.40,1.69)	⨁⨁◯◯ Low

30-day all-cause readmissions (in subgroup 2: usual care as control interventions)	Five RCTs/918 participants	No serious^a^	No serious inconsistency^d^	No serious indirectness	No serious	N/A	0.85(0.60,1.21)	⨁⨁⨁⨁ High


GRADE Working Group grades of evidence. High quality: We are very confident that the true effect lies close to that of the estimate of the effect. Moderate quality: We are moderately confident in the effect estimate: The true effect is likely to be close to the estimate of the effect, but there is a possibility that it is substantially different. Low quality: Our confidence in the effect estimate is limited: The true effect may be substantially different from the estimate of the effect. Very low quality: We have very little confidence in the effect estimate: The true effect is likely to be substantially different from the estimate of effect. Abbreviations: CI, confidence interval; NA: Not applicable; COPD: chronic obstructive pulmonary disease; RCT: Randomized control trial; RR: risk ratio. a: Most information is from studies at low risk of bias or some concerns. Plausible bias is unlikely to seriously alter the results. b: The quality of evidence is downgraded one level for serious imprecision because the 95% CI overlaps the RR of 1.0 but includes important benefit or important harm (RR estimates below 0.5 and above 2.0 are considered clinically important). c: The quality of evidence is downgraded one level for serious inconsistency. Statistical test from pairwise meta-analysis suggests substantial heterogeneity with an I2 value of 63%. d: The quality of evidence for subgroup analysis is not downgraded for inconsistency as there is little variability in results between studies and no suggestion of a subgroup effect. e: The quality of evidence is downgraded two level for very serious imprecision because the 95% CI overlaps the RR of 1.0, but includes important benefit or important harm (RR estimates below 0.5 and above 2.0 are considered clinically important); and the small sample size (less than 200 per group) that may not sufficient to ensure prognostic balance. N/A: Not applicable for publication bias because of less than 10 individual studies.

For secondary outcomes, peri-discharge complex interventions were significantly more effective than controls in reducing 30-day COPD-related readmissions (pooled RR = 0.45, 95% CI: 0.24–0.84, I^2^ = 0%, 4 RCTs) (Appendix 8b), and 3-month all-cause readmissions (pooled RR = 0.74, 95% CI: 0.57–0.95, I^2^ = 0%, 5 RCTs) (Appendix 8c), as supported by high quality evidence (***Table 4***). For other secondary outcomes, moderate quality evidence showed that there was no significant difference between peri-discharge complex interventions and controls in reducing 6-month all-cause readmissions (pooled RR = 0.85, 95% CI: 0.64–1.14, I^2^ = 63%, 4 RCTs) (Appendix 8d, ***Table 4***), and 30-day mortality (pooled RR = 0.35, 95% CI: 0.09–1.34, I^2^ = 0%, 2 RCTs) (Appendix 9, ***Table 4***).

### Sensitivity and subgroup analysis results

Results of sensitivity analysis focusing on three RCTs with low risk of bias showed no significant difference between peri-discharge complex interventions and controls for reducing 30-day all-cause readmissions (pooled RR = 0.80, 95% CI: 0.47–1.38, I^2^ = 27%, 3 RCTs, moderate quality of evidence). Results are presented in Appendix 10 and ***Table 4***.

Results of subgroup analysis based on different comparisons in the control groups were presented in Appendix 11. There was no significance difference in the following subgroups comparisons for reducing 30-day all-cause readmissions: i) peri-discharge complex interventions vs. rehabilitation education (pooled RR = 0.83, 95% CI: 0.40–1.69, I^2^ = 0%, 2 RCTs, low quality of evidence); ii) peri-discharge complex interventions vs. usual care (pooled RR = 0.85, 95% CI: 0.60–1.21, I^2^ = 0%, 5 RCTs, high quality of evidence). Quality of evidence ratings for subgroup analysis are also presented in ***Table 4***.

### Results of NMA

**Figure 2:**
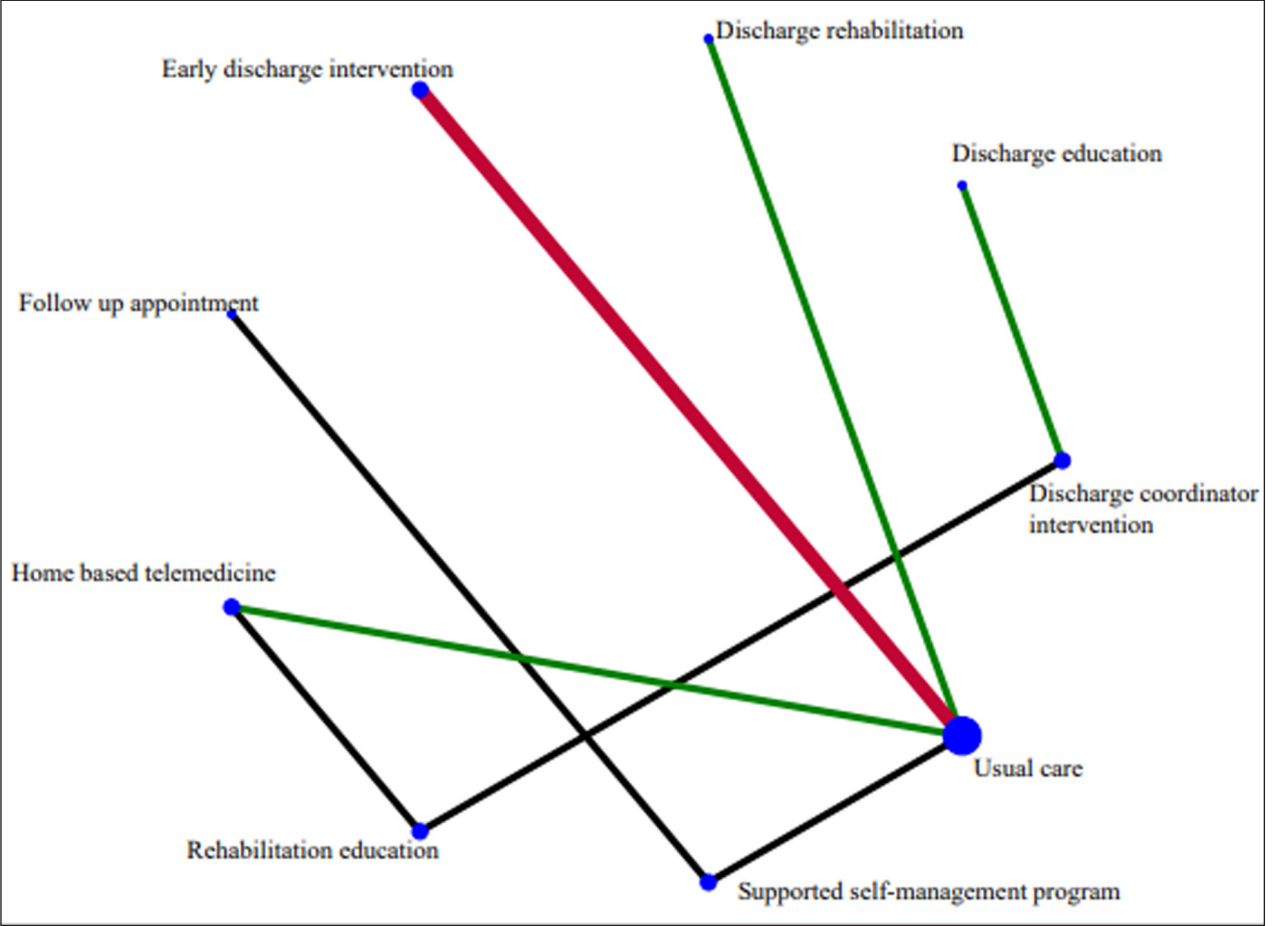
Network plot of comparisons among 8 different peri-discharge complex interventions and usual care in the network meta-analysis for reducing 30-day all-cause readmissions among COPD patients. *Notes*: Peri-discharge complex interventions and usual care are described in Table 1 and 2. Nodes represent theinterventions, node sizes correspond to the number of studies involved, lines connecting nodes represent direct comparisons between pairs of interventions. Width of the lines represents the proportion of the number of trials for each comparison as compared to total number of trials. Line colour indicates different overall risk of bias levels, with red referring to high risk of bias, green referring to low risk of bias, and black referring to some concerns.

For the primary outcome of reducing 30-day all-cause readmissions, the network included nine two-arm trials (***Figure 2***). Size of nodes indicated that usual care was the most common comparator across the included studies. NMA results showed no significant difference among these eight different peri-discharge complex interventions and usual care (Appendix 12).

Using the minimally contextualized framework, all peri-discharge complex interventions were classified into Group 1 based on the network estimates. Their associated quality of evidence and SUCRA results are reported in Appendix 13–14. In Group 1, high certainty of evidence suggested that discharge rehabilitation was not different from usual care, while low certainty of evidence indicated that the remaining seven peri-discharge complex interventions might not differ from usual care (Appendix 15). Results on inconsistency evaluation using the SIDE approach were shown in Appendix 16. As the difference between direct and indirect estimates for each comparison was statistically insignificant, it can be considered that there is no significant inconsistency in this network.

For all secondary outcomes, peri-discharge complex interventions of the included studies did not share a common comparator, hence we could not conduct NMA and evaluate their comparative effectiveness.

## Discussion

### Summary of findings

In this overview of SRs, pairwise meta-analyses showed that there was no significant difference between peri-discharge complex interventions and controls on reducing 30-day all-cause readmissions, 30-day mortality, and 6-month all-cause readmissions among COPD patients. Peri-discharge complex interventions were significantly more effective than controls in reducing 30-day COPD-related readmissions and 3-month all-cause readmissions. As shown in the NMA for 30-day all-cause readmissions, there was no significant difference among the eight peri-discharge complex interventions and usual care. The final classification of complex interventions indicated that discharge rehabilitation was not different from usual care with high certainty of evidence, while the remaining seven peri-discharge complex interventions might be no different from usual care with low certainty of evidence.

### Implications for practice

For the primary outcome of reducing 30-day all-cause readmissions, low certainty of evidence indicated that most complex interventions might be no different from usual care. Nevertheless, our pairwise meta-analysis showed that peri-discharge complex interventions were significantly more effective than controls on reducing 30-day COPD-related readmissions, as supported by high quality evidence. Indeed, COPD-related readmissions accounted for majority of the hospital readmissions within 30 days among COPD patients [53]. Therefore, it can be hypothesized that a reduction in 30-day COPD-related readmissions would potentially relieve the burden of 30-day all-cause readmissions as well, but this claim would require further confirmation.

These effective peri-discharge complex interventions included discharge rehabilitation, supported self-management program, and discharge coordinator intervention. There are several common components among these three peri-discharge complex interventions, namely patient education, self-management, patient-centred discharge instructions, and telephone follow up. These four components might be the core effective elements that play a significant role in contributing to the reduction of 30-day COPD-related readmission. However, potential effectiveness of this four-component package (peri-discharge complex interventions comprising patient education, self-management, patient-centred discharge instructions, and telephone follow up, abbreviated as *Four component peri-discharge complex interventions* hereafter) is likely to be context-sensitive. The decision of implementing such interventions in different health system contexts requires careful deliberations. In the following discussion, we consider selected criteria listed in GRADE Evidence to Decision (EtD) framework [54], which may facilitate the decision-making process.

#### Benefits of implementing the *Four component peri-discharge complex interventions*: patients’ perspective

As a chronic, long-term, and complex illness with multiple systemic effects and co-morbidities, COPD requires a multi-disciplinary and multi-modality approach for optimal management. As suggested by COPD patients, patient education could improve their overall satisfaction with healthcare providers [55], while self-management was an effective way to improve their lifestyle and health behaviour, thereby enhancing their health [56]. With increasing number of COPD patients in the population, self-management was found to be more cost-saving than usual care from the public health system’s perspective [57]. Since symptoms of COPD change frequently due to disease progression [58], patient-centred discharge instructions in the form of a written plan may support patients in responding to their changing symptoms and making appropriate decisions regarding their self-management [59]. On the other hand, telephone follow up by a hospital-based health professional after patient discharge is an effective approach for exchanging information, providing health education, and providing reassurance to patients after discharge [60]. An RCT showed that telephone follow up was effective in enhancing COPD patients’ self-efficacy in managing dyspnea [61]. The synergistic effect of patient education, self-management, patient-centred discharge instructions, and telephone follow up might potentially magnify the individual components effects, since each of them has different mechanisms in improving COPD management.

#### Acceptability of the *Four component peri-discharge complex interventions*

Patient education and self-management are widely accepted by both COPD patients and health professionals. Patients were eager to learn more information about the disease and the self-management approaches [62]. Evaluation of a program comprising of patient education and self-management showed that COPD patients had more confidence in managing their illness after completing the program [63]. These two components have been endorsed in existing guidelines such as the American College of Chest Physicians and Canadian Thoracic Society Guideline, joint American College of Chest Physicians and the American Association of Cardiovascular and Pulmonary Rehabilitation (joint ACCP/AACVPR) evidence-based clinical practice guidelines, and American Thoracic Society/European Respiratory Society statement [64,65,66,67]. Nurses and allied health professionals also showed positive attitudes towards COPD self-management, as it allowed them to manage time and other resources more effectively when compared to usual care provision [68]. Furthermore, a two-round Delphi study conducted in Canada demonstrated that physicians, nurses, and patients have established consensus on including patient-centred discharge instruction as a key component in the discharge care package for COPD patients, to ensure a smooth hospital to community transition, and to reduce risk of disease recurrence and readmission [69]. Telephone follow up was also acceptable to patients, as it is regarded to be much more convenient than face-to-face appointments [70].

#### Feasibility of implementing the *Four component peri-discharge complex interventions*

Despite positive views on benefits and acceptability described above, the implementation of the *Four component peri-discharge complex interventions* could be challenging. Potential barriers towards adopting self-management among COPD patients may include feeling of fear towards increased incidence of exacerbation; perceived lack of capacity to manage exacerbation; and insufficient knowledge on the consequences of inadequate treatment [71,72,73]. To address these barriers, developing tailored educational packages for patients may be an efficient way for empowering COPD patients [74] in improving capacity in self-management. At health professionals’ level, practical constraints, such as inflexible health care infrastructures, excessive workload, and the priority of other duties were considered to reduce their support on the implementation of the *Four component peri-discharge complex interventions* among COPD patients [68,75,76]. These barriers need to be carefully considered prior to implementing the interventions. A clear division of labour and more allocation of resources to health professionals could facilitate their participation in the implementation process.

#### Linkage with integrated care models

There are several existing models of integrated care [77], including but not limited to Model 1: customized integration and disease management model, which describes tailored-made care integration around disease or individuals; Model 2: co-location of care model, which describes joint-venture collaborations relying on co-location of healthcare professionals to achieve coordination of care; Model 3: IT-integrated health care model, which describes the integration relying on information technology approaches; and Model 4: patient integrated health care model, which described integration by engaging patients to coordinate their health information and serve as their own health gatekeeper.

Peri-discharge complex interventions synthesized in this systematic review have more focus on Models 1, 3 and 4. For example, home based telemedicine for COPD patients would feature elements from both IT-integrated health care model and patient integrated health care model. This is because this intervention component involves online monitoring of COPD patients’ physiological data, and such data is used to devise individualized education to patients for enhancing self-care. Further information on different peri-discharge complex interventions components is presented in ***Table 3***.

### Implications for research

The GRADE-ADOLOPMENT approach provides an explicit framework for guiding localized recommendation development process [54]. Future research might invite key stakeholders and conduct a Delphi survey based on the GRADE-ADOLOPMENT framework for achieving consensus on whether the *Four component peri-discharge complex interventions* or usual care should be tailored to address the needs of local health system [54]. In this process, stakeholders can make their decisions by considering problem priority, benefits, harms, equity, acceptability, and feasibility.

Our study showed that most complex interventions might be no different from usual care in reducing 30-day all-cause readmissions, but this conclusion is only supported by low certainty evidence. More trials might therefore be conducted in the future to strengthen the evidence base in this field. However, as a complex intervention, evaluating the effectiveness of peri-discharge complex interventions is known to be challenging [78]. The first challenge is the lack of standardization on the content and fidelity of the peri-discharge complex interventions. Service delivery in different health systems would vary in terms of intensity, frequency as well as combinations of different components, depending on resources available. On the other hand, it is likely that features of the complex interventions would be tailored to patients’ stages of disease, health and social care needs, as well as preferences. The second challenge is inadequate resources allocated for proper evaluation of the peri-discharge complex interventions, which often requires advanced or mixed methods research. Front-line professionals may face time and resources constraints if they are involved in the implementation and evaluation process on top of delivering routine care. The third challenge is to recruit patients who are willing to enrol in trials of different peri-discharge complex interventions as they often expect the best available care when they are in need. Lastly, policy makers may find evaluation of complex intervention too difficult and expensive, which hinder funding support on such trials.

### Strengths and limitations

Strengths of this overview included a comprehensive electronic literature search to identify eligible SRs with an updated search for RCTs; independent literature selection and data extraction; methodological quality and risk of bias assessment conducted independently by two reviewers; application of NMA for comparing three or more interventions simultaneously in a single analysis via the common comparator; and use of GRADE approach for assessing the quality of evidence. Sensitivity analysis focusing only on RCTs with low risk of bias was also conducted, yielding results similar to the primary meta-analysis. This supports the robustness of our findings.

Our findings also have several limitations. Firstly, quality of evidence varied from moderate to low across comparisons in NMA of reducing 30-day all-cause readmissions. Imprecision was the main reason for downgrading the NMA effect estimate. Secondly, with a small number of included RCTs, there was a lack of direct comparisons between many peri-discharge complex interventions, thereby weakening the comprehensiveness of comparisons. As the *Four component peri-discharge complex interventions* might be the core effective interventions in reducing 30-day COPD-related readmissions, more RCTs may be conducted to investigate the comparative effectiveness between the *Four component peri-discharge complex interventions* and well-specified usual care. Lastly, content of usual care as well as individual intervention components may vary according to local practices in different health system contexts. Unclear reporting of intervention content may limit interpretation of our findings. Researchers conducting future RCTs should fully describe usual care and intervention components following the TIDieR guideline [79].

## Conclusion

Peri-discharge complex interventions may not differ from usual care for reducing 30-day all-cause readmissions among COPD patients. Yet, the major cause of 30-day readmissions among these patients is COPD-related. The *Four component peri-discharge complex interventions*, which comprise patient education, self-management, patient-centred discharge instructions, and telephone follow up, seems to be key components in reducing 30-day COPD-related readmissions. This beneficial effect may help to reduce a substantial proportion of readmission. Prior to implementing the *Four component peri-discharge complex interventions*, policymakers from different health system contexts should consider carefully the aspects of problem priority, benefits, harms, equity, acceptability, and feasibility.

## Additional File

The additional file for this article can be found as follows:

10.5334/ijic.6018.s1 Appendices. Appendix 1 to 16.

## References

[1] Vogelmeier CF, Criner GJ, Martinez FJ, Anzueto, A, Barnes, PJ, Bourbeau, J, et al. Global strategy for the diagnosis, management, and prevention of chronic obstructive lung disease 2017 report. GOLD executive summary. American journal of respiratory and critical care medicine. 2017; 195(5): 557–82. DOI: 10.1111/resp.13012 28128970

[2] Institute for Health Metrics and Evaluation (IHME). GBD Results Tool. Seattle, WA: IHME, University of Washington; 2019. [cited 2022 18 January]. Available from: http://ghdx.healthdata.org/gbd-results-tool.

[3] World Health Organisation (WHO). Chronic respiratory diseases. Burden of COPD; 2019. [cited 2022 18 January]. Available from: www.who.int/respiratory/copd/burden/en/.

[4] Fingar K, Washington R. Trends in hospital readmissions for four highvolume conditions, 2009–2013 (Statistical Brief# 196). Rockville, MD: Agency for Healthcare Research and Quality. 26764446

[5] Cakir B, Gammon G. Evaluating readmission rates: how can we improve? Southern medical journal. 2010; 103(11): 1079–83. DOI: 10.1097/SMJ.0b013e3181f20a0f 20890253

[6] Anderson GF, Steinberg EP. Hospital readmissions in the Medicare population. New England Journal of Medicine. 1984; 311(21): 1349–53. DOI: 10.1056/NEJM198411223112105 6436703

[7] Vest JR, Gamm LD, Oxford BA, Gonzalez MI, Slawson KM. Determinants of preventable readmissions in the United States: a systematic review. Implementation Science. 2010; 5(1): 88. DOI: 10.1186/1748-5908-5-88 21083908 PMC2996340

[8] Jencks SF, Williams MV, Coleman EA. Rehospitalizations among patients in the Medicare fee-for-service program. New England Journal of Medicine. 2009; 360(14): 1418–28. DOI: 10.1056/NEJMsa0803563 19339721

[9] World Health Organization. Do current discharge arrangements from inpatient hospital care for the elderly reduce readmission rates, the length of inpatient stay or mortality, or improve health status? 2005. [cited 2022 18 January]. Available from: https://www.euro.who.int/en/health-topics/Life-stages/healthy-ageing/publications/pre-2009/do-current-discharge-arrangements-from-inpatient-hospital-care-for-the-elderly-reduce-readmission-rates,-the-length-of-inpatient-stay-or-mortality,-or-improve-health-stat.

[10] Pittsburgh Regional Health Initiative. PRHI readmission: Brief 1: overview of six target chronic diseases. Pittsburgh: Pittsburgh Regional Health Initiative; 2010 Mar. [cited 2022 18 January]. Available from: www.chqpr.org/downloads/PRHI_ReadmissionBrief_ChronicDisease_June2010.pdf.

[11] Chan FW, Wong FY, Yam CH, Cheung WL, Wong EL, Leung MC, et al. Risk factors of hospitalization and readmission of patients with COPD in Hong Kong population: analysis of hospital admission records. BMC health services research. 2011; 11(1): 186. DOI: 10.1186/1472-6963-11-186 21831287 PMC3162881

[12] Carneiro R, Sousa C, Pinto A, Almeida F, Oliveira JR, Rocha N. Risk factors for readmission after hospital discharge in chronic obstructive pulmonary disease. The role of quality of life indicators. Revista Portuguesa de Pneumologia (English Edition). 2010; 16(5): 759–77. DOI: 10.1016/S2173-5115(10)70076-2 20927493

[13] Halpin DM, Miravitlles M, Metzdorf N, Celli B. Impact and prevention of severe exacerbations of COPD: a review of the evidence. International journal of chronic obstructive pulmonary disease. 2017; 12: 2891–908. DOI: 10.2147/COPD.S139470 29062228 PMC5638577

[14] Glynn N, Bennett K, Silke B. Emergency medical readmission: long-term trends and impact on mortality. Clinical Medicine. 2011; 11(2): 114–8. DOI: 10.7861/clinmedicine.11-2-114 21526689 PMC5922729

[15] de Miguel-Díez J, Jiménez-García R, Hernández-Barrera V, Carrasco-Garrido P, Maestu LP, García LR, et al. Readmissions following an initial hospitalization by COPD exacerbation in S pain from 2006 to 2012. Respirology. 2016; 21(3): 489–96. DOI: 10.1111/resp.12705 26648085

[16] Oddone EZ, Weinberger M, Horner M, Mengel C, Goldstein F, Ginier P, et al. Classifying general medicine readmissions. Journal of general internal medicine. 1996; 11(10): 597–607. DOI: 10.1007/BF02599027 8945691

[17] Press VG, Au DH, Bourbeau J, Dransfield MT, Gershon AS, Krishnan JA, et al. Reducing chronic obstructive pulmonary disease hospital readmissions. An official American Thoracic Society workshop report. Annals of the American Thoracic Society. 2019; 16(2): 161–70. DOI: 10.1513/AnnalsATS.201811-755WS 30707066 PMC6812156

[18] Stone RA, Holzhauer-Barrie J, Lowe D, McMillan V, Saleem Khan M, Searle L, et al. COPD: Who cares when it matters most? National Chronic Obstructive Pulmonary Disease (COPD) Audit Programme: Outcomes from the clinical audit of COPD exacerbations admitted to acute units in England 2014. National supplementary report. 2017.

[19] Stone JL, Hoffman G. Medicare hospital readmissions: Issues, policy options and PPACA. Washington, DC: Congressional Research Service; 2010.

[20] The Global Initiative for Chronic Obstructive Lung Disease (GOLD). Global strategy for the diagnosis, management, and prevention of chronic obstructive lung disease (2020 report). Global Initiative for Chronic Obstructive Lung Disease; 2020. [cited 2022 18 January]. Available from: https://goldcopd.org/wp-content/uploads/2019/12/GOLD-2020-FINAL-ver1.2-03Dec19_WMV.pdf.

[21] Pedersen PU, Ersgard KB, Soerensen TB, Larsen P. Effectiveness of structured planned post discharge support to patients with chronic obstructive pulmonary disease for reducing readmission rates: A systematic review. JBI Database of Systematic Reviews and Implementation Reports. 2017; 15(8): 2060–86. DOI: 10.11124/JBISRIR-2016-003045 28800056

[22] Echevarria C, Brewin K, Horobin H, Bryant A, Corbett S, Steer J, et al. Early Supported Discharge/Hospital At Home For Acute Exacerbation of Chronic Obstructive Pulmonary Disease: A Review and Meta-Analysis. COPD. 2016; 13(4): 523–33. DOI: 10.3109/15412555.2015.1067885 26854816

[23] Ryrso CK, Godtfredsen NS, Kofod LM, Lavesen M, Mogensen L, Tobberup R, et al. Lower mortality after early supervised pulmonary rehabilitation following COPD-exacerbations: a systematic review and meta-analysis. BMC pulmonary medicine. 2018; 18(1): 154. DOI: 10.1186/s12890-018-0718-1 30219047 PMC6139159

[24] Naylor MD, Aiken LH, Kurtzman ET, Olds DM, Hirschman KB. The importance of transitional care in achieving health reform. Health affairs. 2011; 30(4): 746–54. DOI: 10.1377/hlthaff.2011.0041 21471497

[25] Contandriopoulos AP, Université de Montréal. Groupe de recherche interdisciplinaire en santé. The integration of health care: dimensions and implementation. GRIS, Université de Montréal; 2004.

[26] Nolte E, Pitchforth E. Policy Summary 11: What is the evidence on the economic impacts of integrated care? Copenhagen: European Observatory on Health Systems and Policies; 2014. [cited 2022 18 January]. Available from: https://www.euro.who.int/data/assets/pdf_file/0019/251434/What-is-the-evidence-on-the-economic-impacts-of-integrated-care.pdf.

[27] Harrison SL, Janaudis-Ferreira T, Brooks D, Desveaux L, Goldstein RS. Self-management following an acute exacerbation of COPD: A systematic review. Chest. 2015; 147(3): 646–61. DOI: 10.1378/chest.14-1658 25340578

[28] Jeppesen E, Brurberg KG, Vist GE, Wedzicha JA, Wright JJ, Greenstone M, et al. Hospital at home for acute exacerbations of chronic obstructive pulmonary disease. The Cochrane database of systematic reviews. 2012; 5: CD003573. DOI: 10.1002/14651858.CD003573.pub2 PMC11622732 22592692

[29] Hutton B, Salanti G, Caldwell DM, Chaimani A, Schmid CH, Cameron C, et al. The PRISMA extension statement for reporting of systematic reviews incorporating network meta-analyses of health care interventions: checklist and explanations. Annals of internal medicine. 2015; 162(11): 777–84. DOI: 10.7326/M14-2385 26030634

[30] Higgins JP, Thomas J, Chandler J, Cumpston M, Li T, Page MJ, et al, editors. Cochrane handbook for systematic reviews of interventions. John Wiley & Sons; 2019 Sep 23. DOI: 10.1002/9781119536604 PMC10284251 31643080

[31] Martinic MK, Pieper D, Glatt A, Puljak L. Definition of a systematic review used in overviews of systematic reviews, meta-epidemiological studies and textbooks. BMC medical research methodology. 2019; 19(1): 203. DOI: 10.1186/s12874-019-0855-0 31684874 PMC6829801

[32] Craig P, Dieppe P, Macintyre S, Michie S, Nazareth I, Petticrew M. Developing and evaluating complex interventions: the new Medical Research Council guidance. BMJ. 2008: 337. DOI: 10.1136/bmj.a1655 PMC2769032 18824488

[33] Horwitz LI, Grady JN, Dorsey KB, Zhang W, Keenan M, Keshawarz A, et al. 2014 Measure Updates and Specification Report: Hospital-Wide All-Cause Unplanned Readmission–Version 3.0. New Haven: Yale New Haven Health Services Corporation/Center for Outcomes Research & Evaluation; 2014 July. [cited 2022 18 January]. Available from: https://altarum.org/sites/default/files/uploaded-publication-files/Rdmsn_Msr_Updts_HWR_0714_0.pdf.

[34] Centers for Medicare & Medicaid Services. Hospital-Wide All-Cause Unplanned Readmission Measure (HWR). Baltimore: Centers for Medicare & Medicaid Services. 2012. [cited 2022 18 January]. Available from: https://www.cms.gov/Medicare/Quality-Initiatives-Patient-Assessment-Instruments/HospitalQualityInits/Measure-Methodology.

[35] Mcllvennan CK, Eapen ZJ, Allen LA. Hospital readmissions reduction program. Circulation. 2015; 131(20): 1796–803. DOI: 10.1161/CIRCULATIONAHA.114.010270 25986448 PMC4439931

[36] Soler-Cataluña JJ, Martínez-García MA, Román Sánchez P, Salcedo E, Navarro M, Ochando R. Severe acute exacerbations and mortality in patients with chronic obstructive pulmonary disease. Thorax. 2005; 60(11): 925–31. DOI: 10.1136/thx.2005.040527 16055622 PMC1747235

[37] Miravitlles M, Ferrer M, Pont A, Zalacain R, Alvarez-Sala JL, Masa F, et al. Effect of exacerbations on quality of life in patients with chronic obstructive pulmonary disease: a 2 year follow up study. Thorax. 2004; 59(5): 387–95. DOI: 10.1136/thx.2003.008730 15115864 PMC1746989

[38] Shea BJ, Reeves BC, Wells G, Thuku M, Hamel C, Moran J, et al. AMSTAR 2: a critical appraisal tool for systematic reviews that include randomised or non-randomised studies of healthcare interventions, or both. BMJ. 2017; 358: j4008. DOI: 10.1136/bmj.j4008 28935701 PMC5833365

[39] Sterne JAC, Savovic J, Page MJ, Elbers RG, Blencowe NS, Boutron I, et al. RoB 2: a revised tool for assessing risk of bias in randomised trials. BMJ. 2019; 366: l4898. DOI: 10.1136/bmj.l4898 31462531

[40] Schünemann HJ, Higgins JP, Vist GE, Glasziou P, Akl EA, Skoetz N, et al. Completing ‘Summary of findings’ tables and grading the certainty of the evidence. In: Higgins JPT, Thomas J, Chandler J, Cumpston M, Li T, Page MJ, et al. (eds.), Cochrane Handbook for systematic reviews of interventions. London: Cochrane; 2008.

[41] Salanti G, Del Giovane C, Chaimani A, Caldwell DM, Higgins JPT. Evaluating the quality of evidence from a network meta-analysis. PloS one. 2014; 9(7): e99682. DOI: 10.1371/journal.pone.0099682 24992266 PMC4084629

[42] Hansen LO, Young RS, Hinami K, Leung A, Williams MV. Interventions to reduce 30-day rehospitalization: a systematic review. Ann Intern Med. 2011; 155(8): 520–8. DOI: 10.7326/0003-4819-155-8-201110180-00008 22007045

[43] Salanti G. Indirect and mixed-treatment comparison, network, or multiple-treatments meta-analysis: many names, many benefits, many concerns for the next generation evidence synthesis tool. Res Synth Methods. 2012; 3(2): 80–97. DOI: 10.1002/jrsm.1037 26062083

[44] Higgins JPT, Thompson SG, Deeks JJ, Altman DG. Measuring inconsistency in meta-analyses. BMJ. 2003; 327(7414): 557–60. DOI: 10.1136/bmj.327.7414.557 12958120 PMC192859

[45] Lu G, Ades AE. Combination of direct and indirect evidence in mixed treatment comparisons. Statistics in medicine. 2004; 23(20): 3105–24. DOI: 10.1002/sim.1875 15449338

[46] Mills EJ, Thorlund K, Ioannidis JP. Demystifying trial networks and network meta-analysis. BMJ. 2013; 346: f2914. DOI: 10.1136/bmj.f2914 23674332

[47] Chaimani A, Higgins JP, Mavridis D, Spyridonos P, Salanti G. Graphical tools for network meta-analysis in STATA. PLoS One. 2013; 8(10): e76654. DOI: 10.1371/journal.pone.0076654 24098547 PMC3789683

[48] White IR. Multivariate random-effects meta-regression: updates to mvmeta. The Stata Journal. 2011; 11(2): 255–70. DOI: 10.1177/1536867X1101100206

[49] Salanti G, Ades AE, Ioannidis JPA. Graphical methods and numerical summaries for presenting results from multiple-treatment meta-analysis: an overview and tutorial. Journal of clinical epidemiology. 2011; 64(2): 163–71. DOI: 10.1016/j.jclinepi.2010.03.016 20688472

[50] Dias S, Welton NJ, Caldwell DM, Ades AE. Checking consistency in mixed treatment comparison meta-analysis. Statistics in Medicine. 2010; 29(7–8): 932–44. DOI: 10.1002/sim.3767 20213715

[51] Chaimani A, Caldwell DM, Li T, Higgins JP, Salanti G. Undertaking network meta-analyses. In: Higgins JPT, Thomas J, Chandler J, Cumpston M, Li T, Page MJ, et al. (eds.), Cochrane Handbook for systematic reviews of interventions. 2019; 285–320. London: Cochrane. DOI: 10.1002/9781119536604.ch11

[52] Brignardello-Petersen R, Florez ID, Izcovich A, Santesso N, Hazlewood G, Alhazanni W, et al. GRADE approach to drawing conclusions from a network meta-analysis using a minimally contextualised framework. BMJ. 2020; 371. DOI: 10.1136/bmj.m3900 33177059

[53] AlHafidh OZ, Sidhu JS, Virk J, Patel N, Patel Z, Gayam V, et al. Incidence, Predictors, Causes, and Cost of 30-Day Hospital Readmission in Chronic Obstructive Pulmonary Disease Patients Undergoing Bronchoscopy. Cureus. 2020; 12(6): e8607. DOI: 10.7759/cureus.8607 32550091 PMC7294856

[54] Schünemann HJ, Wiercioch W, Brozek J, Etxeandia-Ikobaltzeta I, Mustafa RA, Manja V, et al. GRADE Evidence to Decision (EtD) frameworks for adoption, adaptation, and de novo development of trustworthy recommendations: GRADE-ADOLOPMENT. Journal of clinical epidemiology. 2017; 81: 101–10. DOI: 10.1016/j.jclinepi.2016.09.009 27713072

[55] Gallefoss F, Bakke P. Patient satisfaction with healthcare in asthmatics and patients with COPD before and after patient education. Respiratory medicine. 2000; 94(11): 1057–64. DOI: 10.1053/rmed.2000.0886 11127492

[56] Bourbeau J, Nault D, Dang-Tan T. Self-management and behaviour modification in COPD. Patient education and counseling. 2004; 52(3): 271–7. DOI: 10.1016/S0738-3991(03)00102-2 14998597

[57] Bourbeau J, Collet J-P, Schwartzman K, Ducruet T, Nault D, Bradley C. Economic benefits of self-management education in COPD. Chest. 2006; 130(6): 1704–11. DOI: 10.1378/chest.130.6.1704 17166985

[58] Global Initiative for Chronic Obstructive Lung Disease. Global strategy for the diagnosis, management and prevention of Chronic Obstructive Pulmonary Disease (2022 Report). [cited 2022 18 January]. Available from: https://goldcopd.org/2022-gold-reports-2/

[59] Effing TW, Vercoulen JH, Bourbeau J, Trappenburg J, Lenferink A, Cafarella P, et al. Definition of a COPD self-management intervention: International Expert Group consensus. European Respiratory Journal. 2016; 48(1): 46–54. DOI: 10.1183/13993003.00025-2016 27076595

[60] Mistiaen P, Poot E. Telephone follow-up, initiated by a hospital-based health professional, for postdischarge problems in patients discharged from hospital to home. Cochrane Database of Systematic Reviews. 2006; (4). DOI: 10.1002/14651858.CD004510.pub3 PMC6823218 17054207

[61] Liu WT, Wang CH, Lin HC, Lin SM, Lee KY, Lo YL, et al. Efficacy of a cell phone-based exercise programme for COPD. European Respiratory Journal. 2008; 32(3): 651–9. DOI: 10.1183/09031936.00104407 18508824

[62] Sandelowsky H, Krakau I, Modin S, Ställberg B, Nager A. COPD patients need more information about self-management: a cross-sectional study in Swedish primary care. Scandinavian journal of primary health care. 2019; 37(4): 459–67. DOI: 10.1080/02813432.2019.1684015 31694439 PMC6883432

[63] Apps LD, Harrison SL, Mitchell KE, Williams JE, Hudson N, Singh SJ. A qualitative study of patients’ experiences of participating in SPACE for COPD: a Self-management Programme of Activity, Coping and Education. ERJ open research. 2017; 3(4). DOI: 10.1183/23120541.00017-2017 PMC5703355 29204434

[64] Criner GJ, Bourbeau J, Diekemper RL, Ouellette DR, Goodridge D, Hernandez P, et al. Prevention of acute exacerbations of COPD: American college of chest physicians and Canadian thoracic society guideline. Chest. 2015; 147(4): 894–942. DOI: 10.1378/chest.14-1676 25321320 PMC4388124

[65] Camp PG, Hernandez P, Bourbeau J, Kirkham A, Debigare R, Stickland MK, et al. Pulmonary rehabilitation in Canada: a report from the Canadian thoracic society COPD clinical assembly. Canadian respiratory journal. 2015; 22(3): 147–52. DOI: 10.1155/2015/369851 25848802 PMC4470547

[66] Ries AL, Bauldoff GS, Carlin BW, Casaburi R, Emery CF, Mahler DA, et al. Pulmonary rehabilitation: joint ACCP/AACVPR evidence-based clinical practice guidelines. Chest. 2007; 131(5): 4S–42S. DOI: 10.1378/chest.06-2418 17494825

[67] Spruit MA, Singh SJ, Garvey C, ZuWallack R, Nici L, Rochester C, et al. An official American Thoracic Society/European Respiratory Society statement: key concepts and advances in pulmonary rehabilitation. American journal of respiratory and critical care medicine. 2013; 188(8): e13-e64. DOI: 10.1164/rccm.201309-1634ST 24127811

[68] Young HML, Apps LD, Harrison SL, Johnson-Warrington VL, Hudson N, Singh SJ. Important, misunderstood, and challenging: a qualitative study of nurses’ and allied health professionals’ perceptions of implementing self-management for patients with COPD. International journal of chronic obstructive pulmonary disease. 2015; 10: 1043–52. DOI: 10.2147/COPD.S78670 26082628 PMC4461084

[69] Ospina MB, Michas M, Deuchar L, Leigh R, Bhutani M, Rowe BH, et al. Development of a patient-centred, evidence-based and consensus-based discharge care bundle for patients with acute exacerbation of chronic obstructive pulmonary disease. BMJ open respiratory research. 2018; 5(1): e000265. DOI: 10.1136/bmjresp-2017-000265 PMC5812389 29468074

[70] Walters JA, Cameron-Tucker H, Courtney-Pratt H, Nelson M, Robinson A, Scott J, et al. Supporting health behaviour change in chronic obstructive pulmonary disease with telephone health-mentoring: insights from a qualitative study. BMC family practice. 2012; 13(1): 55. DOI: 10.1186/1471-2296-13-55 22694996 PMC3411441

[71] Korpershoek YJ, Vervoort SC, Nijssen LI, Trappenburg JC, Schuurmans MJ. Factors influencing exacerbation-related self-management in patients with COPD: a qualitative study. International Journal of Chronic Obstructive Pulmonary Disease. 2016; 11: 2977–90. DOI: 10.2147/COPD.S116196 27932877 PMC5135062

[72] Sohanpal R, Seale C, Taylor SJ. Learning to manage COPD: a qualitative study of reasons for attending and not attending a COPD-specific self-management programme. Chronic Respiratory Disease. 2012; 9(3): 163–74. DOI: 10.1177/1479972312444630 22637746

[73] Hernandez P, Balter M, Bourbeau J, Hodder R. Living with chronic obstructive pulmonary disease: a survey of patients’ knowledge and attitudes. Respiratory medicine. 2009; 103(7): 1004–12. DOI: 10.1016/j.rmed.2009.01.018 19269150

[74] Wilson JS, O’Neill B, Reilly J, MacMahon J, Bradley JM. Education in pulmonary rehabilitation: the patient’s perspective. Archives of physical medicine and rehabilitation. 2007; 88(12): 1704–9. DOI: 10.1016/j.apmr.2007.07.040 18047889

[75] Lehn SF, Thuesen J, Bunkenborg G, Zwisler A-D, Rod MH. Implementation between text and work—a qualitative study of a readmission prevention program targeting elderly patients. Implementation Science. 2018; 13(1): 38. DOI: 10.1186/s13012-018-0730-0 29490671 PMC5831845

[76] Rask KJ, Hodge J, Kluge L. Impact of Contextual Factors on Interventions to Reduce Acute Care Transfers II Implementation and Hospital Readmission Rates. Journal of the American Medical Directors Association. 2017; 18(11): 991.e11–991.e15. DOI: 10.1016/j.jamda.2017.08.002 28967602

[77] Burns, LR, Pauly, MV. Integrated delivery networks: A detour on the road tointegrated health care? Health Affairs. 2002; 21(4): 128–43. DOI: 10.1377/hlthaff.21.4.128 12117123

[78] Datta J, Petticrew M. Challenges to evaluating complex interventions: a content analysis of published papers. BMC public health. 2013; 13(1): 1–18. DOI: 10.1186/1471-2458-13-568 23758638 PMC3699389

[79] Hoffmann TC, Glasziou PP, Boutron I, Milne R, Perera R, Moher D, et al. Better reporting of interventions: template for intervention description and replication (TIDieR) checklist and guide. BMJ. 2014; 348: g1687. DOI: 10.1136/bmj.g1687 24609605

