# Supplementary material for: Effectiveness of Peri-Discharge Complex Interventions for Reducing 30-Day Readmissions among COPD Patients: Overview of Systematic Reviews and Network Meta-Analysis
Source: Int J Integr Care. 2022 Feb 3;22(1):7. doi: 10.5334/ijic.6018 (PMC8815439; doi:10.5334/ijic.6018)
Supplement: Appendices. — Appendix 1 to 16. [file ijic-22-1-6018-s1.pdf]

Appendix 1a Search strategies and results for systematic reviews (SRs) on interventions for reducing hospital readmissions

(i) MEDLINE from inception to August 30, 2019

#	Search Statement	Results
1	patient discharge/ or "hospital discharge".mp.	45005
2	1 and patient care planning/	749
3	(readmit* or readmission*).mp. or patient readmission/ or (re adj (admit* or admission* or hospital*)).mp. or rehospital*.mp. or postdischarge*.mp. or (post adj discharge).mp.	38121
4	3 and (intervention*.ti,ab. or interventional study.pt.)	6907
5	(discharge planning or "discharge instruction*" or "individuali?ed plan").mp.	3356
6	(aftercare or after care or ((discharge or care) adj2 bundle*)).mp.	12155
7	("30" adj day*1).mp.	87847
8	patient care management/ or primary health care/ or patient education as topic/ or "patient education".mp. or "care plan*1".mp. or "care pathway*".mp.	177131
9	continuity of patient care/ or geriatric assessment/ or mobility limitation/ or exp rehabilitation/ or exp physical therapy modalities/	334497
10	(telephone or telemonitor* or telemedicine).mp. or home care services/ or house calls/ or support*.mp. or physical fitness.mp. or exercise/ or exercise therapy/ or activities of daily living/ or self care/	9352029
11	((care or case or self) adj manage*).mp. or disability evaluation/ or "social work*".mp. or primary care team/ or community health services/ or "care transition".mp. or counseling.mp. or coaching.mp. or remind*.mp. or health services for the elderly/	256937
12	"Appointments and Schedules"/	8647
13	risk reduction behavior/ or risk assessment/ or risk factors/ or health knowledge attitudes/ or patient satisfaction/ or "home visit*".mp. or home nursing.mp. or pharmacist*.mp. or medication reconciliation/ or (hospital adj2 home).mp. or barrier*.mp. or family practice/ or physicians, family/ or "follow-up".mp.	2393751
14	(socioeconomic factors or literacy).mp. or marital status/ or "social adj support".mp.	174916
15	1 and 3 and (intervention*.mp. or patient care planning/)	1727
16	or/4-14	11210514
17	(readmit* or readmission*).ti. or patient readmission/ or (re adj (admit* or admission* or hospital*)).ti. or rehospital*.ti. or postdischarge*.ti. or (post adj discharge).ti.	16482
18	3 and 16	29715

#	Search Statement	Results
19	17 and 18	13124
20	meta analysis.mp,pt. or review.pt. or search:.tw.	2618275
21	19 and 20	778

(ii) EMBASE from inception to August 30, 2019

#	Search Statement	Results
1	patient discharge/ or "hospital discharge".mp.	126441
2	1 and patient care planning/	695
3	(readmit* or readmission*).mp. or patient readmission/ or (re adj (admit* or admission* or hospital*)).mp. or rehospital*.mp. or postdischarge*.mp. or (post adj discharge).mp.	89129
4	3 and (intervention*.ti,ab. or interventional study.pt.)	16489
5	(discharge planning or "discharge instruction*" or "individuali?ed plan").mp.	5691
6	(aftercare or after care or ((discharge or care) adj2 bundle*)).mp.	11680
7	("30" adj day*1).mp.	168705
8	patient care management/ or primary health care/ or patient education as topic/ or "patient education".mp. or "care plan*1".mp. or "care pathway*".mp.	444904
9	continuity of patient care/ or geriatric assessment/ or mobility limitation/ or exp rehabilitation/ or exp physical therapy modalities/	693726
10	(telephone or telemonitor* or telemedicine).mp. or home care services/ or house calls/ or support*.mp. or physical fitness.mp. or exercise/ or exercise therapy/ or activities of daily living/ or self care/	2385471
11	((care or case or self) adj manage*).mp. or disability evaluation/ or "social work*".mp. or primary care team/ or community health services/ or "care transition".mp. or counseling.mp. or coaching.mp. or remind*.mp. or health services for the elderly/	462427
12	"Appointments and Schedules"/	40196
13	risk reduction behavior/ or risk assessment/ or risk factors/ or health knowledge attitudes/ or patient satisfaction/	1937066

#	Search Statement	Results
	or "home visit*".mp. or home nursing.mp. or pharmacist*.mp. or medication reconciliation/ or (hospital adj2 home).mp. or barrier*.mp. or family practice/ or physicians, family/ or "follow-up".mp.	
14	(socioeconomic factors or literacy).mp. or marital status/ or "social adj support".mp.	83042
15	1 and 3 and (intervention*.mp. or patient care planning/)	3861
16	4 or 5 or 6 or 7 or 8 or 9 or 10or 11 or 12 or 13 or 14	5097296
17	3 and 16 and randomized controlled trial/	3119
18	15 or 17	6396
19	(readmit* or readmission*).ti. or patient readmission/ or (re adj (admit* or admission* or hospital*)).ti. or rehospital*.ti. or postdischarge*.ti. or (post adj discharge).ti. 17 and 18	50592
20	hospital readmission/	56176
21	hospital readmission*.mp.	57741
22	19 or 20 or 21	60082
23	meta-analys:.mp. or search:.tw. or review.pt.	2984596
24	15 and 16 and 22 and 23	238

(iii) Cochrane Database of Systematic Reviews （CDSR）from inception to August 30, 2019

("re- admission" OR "re- admit*" OR readmission* OR readmit*) AND (planning OR intervention*) (60)

(iv) Global Health from inception to August 30, 2019

#	Search Statement	Results
1	patient discharge/ or "hospital discharge".mp.	3904
2	1 and patient care planning/	0
3	(readmit* or readmission*).mp. or patient readmission/ or (re adj (admit* or admission* or hospital*)).mp. or rehospital*.mp. or postdischarge*.mp. or (post adj discharge).mp.	3928
4	3 and (intervention*.ti,ab. or interventional study.pt.)	688
5	(discharge planning or "discharge instruction*" or "individuali?ed plan").mp.	167
6	(aftercare or after care or ((discharge or care) adj2 bundle*)).mp.	437
7	("30" adj day*1).mp.	20922
8	patient care management/ or primary health care/ or patient education as topic/ or "patient education".mp. or "care plan*1".mp. or "care pathway*".mp.	14751
9	continuity of patient care/ or geriatric assessment/ or mobility limitation/ or exp rehabilitation/ or exp physical therapy modalities/	1155
10	(telephone or telemonitor* or telemedicine).mp. or home care services/ or house calls/ or support*.mp. or physical fitness.mp. or exercise/ or exercise therapy/ or activities of daily living/ or self care/	228526
11	((care or case or self) adj manage*).mp. or disability evaluation/ or "social work*".mp. or primary care team/ or community health services/ or "care transition".mp. or counseling.mp. or coaching.mp. or remind*.mp. or health services for the elderly/	28493
12	"Appointments and Schedules"/	0
13	risk reduction behavior/ or risk assessment/ or risk factors/ or health knowledge attitudes/ or patient satisfaction/ or "home visit*".mp. or home nursing.mp. or pharmacist*.mp. or medication reconciliation/ or (hospital adj2 home).mp. or barrier*.mp. or family practice/ or physicians, family/ or "follow-up".mp.	361046
14	(socioeconomic factors or literacy).mp. or marital status/ or "social adj support".mp.	10092
15	1 and 3 and (intervention*.mp. or patient care planning/)	78

#	Search Statement	Results
16	or/4-14	595139
17	(readmit* or readmission*).ti. or patient readmission/ or (re adj (admit* or admission* or hospital*)).ti. or rehospital*.ti. or postdischarge*.ti. or (post adj discharge).ti.	670
18	3 and 16	2464
19	17 and 18	503
20	limit 19 to abstracts	500
21	review.mp. [mp=abstract, title, original title, broad terms, heading words, identifiers, cabicodes]	202588
22	19 and 21	43

(v) AMED (Allied and Complementary Medicine) from inception to August 30, 2019

#	Search Statement	Results
1	patient discharge/ or "hospital discharge".mp.	980
2	1 and patient care planning/	17
3	(readmit* or readmission*).mp. or patient readmission/ or (re adj (admit* or admission* or hospital*)).mp. or rehospital*.mp. or postdischarge*.mp. or (post adj discharge).mp.	604
4	3 and (intervention*.ti,ab. or interventional study.pt.)	179
5	(discharge planning or "discharge instruction*" or "individuali?ed plan").mp.	159
6	(aftercare or after care or ((discharge or care) adj2 bundle*)).mp.	33899
7	("30" adj day*1).mp.	609
8	patient care management/ or primary health care/ or patient education as topic/ or "patient education".mp. or "care plan*1".mp. or "care pathway*".mp.	3932
9	continuity of patient care/ or geriatric assessment/ or mobility limitation/ or exp rehabilitation/ or exp physical therapy modalities/	74943

#	Search Statement	Results
10	(telephone or telemonitor* or telemedicine).mp. or home care services/ or house calls/ or support*.mp. or physical fitness.mp. or exercise/ or exercise therapy/ or activities of daily living/ or self care/	51442
11	((care or case or self) adj manage*).mp. or disability evaluation/ or "social work*".mp. or primary care team/ or community health services/ or "care transition".mp. or counseling.mp. or coaching.mp. or remind*.mp. or health services for the elderly/	15153
12	"Appointments and Schedules"/	0
13	risk reduction behavior/ or risk assessment/ or risk factors/ or health knowledge attitudes/ or patient satisfaction/ or "home visit*".mp. or home nursing.mp. or pharmacist*.mp. or medication reconciliation/ or (hospital adj2 home).mp. or barrier*.mp. or family practice/ or physicians, family/ or "follow-up".mp.	21957
14	(socioeconomic factors or literacy).mp. or marital status/ or "social adj support".mp.	1477
15	1 and 3 and (intervention*.mp. or patient care planning/)	47
16	or/4-14	140423
17	(readmit* or readmission*).ti. or patient readmission/ or (re adj (admit* or admission* or hospital*)).ti. or rehospital*.ti. or postdischarge*.ti. or (post adj discharge).ti.	155
18	3 and 16	551
19	17 and 18	136
20	limit 19 to abstracts	95
21	review.mp. [mp=abstract, heading words, title]	18135
22	19 and 21	4

Appendix 1b Search strategies and results for additional randomized controlled trials (RCTs) on interventions for reducing 30-day hospital readmission among patients with COPD

(i) MEDLINE from database inception to June 16, 2020

#	Search Statement	Results
1	(readmit* or readmission*).mp. or patient readmission/ or (re adj (admit* or admission* or hospital*)).mp. or rehospital*.mp. or postdischarge*.mp. or (post adj discharge).mp.	42232
2	Lung Diseases, Obstructive/	18179
3	exp Pulmonary Disease, Chronic Obstructive/	55310
4	emphysema$.mp.	33221
5	(chronic$ adj3 bronchiti$).mp.	10965
6	(obstruct$ adj3 (pulmonary or lung$ or airway$ or airflow$ or bronch$ or respirat$)).mp.	107235
7	COPD.mp.	39361
8	COAD.mp.	280
9	COBD.mp.	18
10	AECB.mp.	211
11	2 or 3 or 4 or 5 or 6 or 7 or 8 or 9 or 10	143259
12	1 and 11	1439
13	randomized controlled trial.pt. or randomized.mp. or placebo.mp.	822539
14	12 and 13	193

(ii) EMBASE from database inception to June 16, 2020

#	Search Statement	Results
1	(readmit* or readmission*).mp. or patient readmission/ or (re adj (admit* or admission* or hospital*)).mp. or rehospital*.mp. or postdischarge*.mp. or (post adj discharge).mp.	98725
2	Lung Diseases, Obstructive/	80961
3	exp Pulmonary Disease, Chronic Obstructive/	132199
4	emphysema$.mp.	46069

#	Search Statement	Results
5	(chronic$ adj3 bronchiti$).mp.	18363
6	(obstruct$ adj3 (pulmonary or lung$ or airway$ or airflow$ or bronch$ or respirat$)).mp.	205544
7	COPD.mp.	89204
8	COAD.mp.	520
9	COBD.mp.	23
10	AECB.mp.	287
11	2 or 3 or 4 or 5 or 6 or 7 or 8 or 9 or 10	262449
12	1 and 11	4793
13	random:.tw. or placebo:.mp. or double-blind:.tw.	1794480
14	12 and 13	542

(iii) Cochrane CENTRAL database inception to June 16, 2020

#	Search Statement	Results
1	MeSH descriptor: [Pulmonary Disease, Chronic Obstructive] explode all trees	5616
2	MeSH descriptor: [Bronchitis, Chronic] explode all trees	165
3	(obstruct*) near3 (pulmonary or lung* or airway* or airflow* or bronch* or respirat*)	91
4	COPD OR COAD OR COBD	16277
5	#1 or #2 or #3 or #4	17358
6	MeSH descriptor: [Patient Readmission] explode all trees	1015
7	(readmission or readmitted or re-admission or re-admitted):ti,ab,kw (Word variations have been searched)	6966
8	(rehospitali?ation* or re-hospitali?ation* or rehospitali?ed or re-hospitali?ed):ti,ab,kw (Word variations have been searched)	2328
9	#6 or #7 or #8	8298
10	#5 and #9 in Trials	389

Appendix 2 Details of literature selection, data extraction, methodological quality assessments, risk of bias assessment, and quality of evidence rating

First, we screened title and abstract to assess preliminary eligibility. Full texts of potentially eligible citations were further evaluated for final inclusion or exclusion. After literature selection, we generated a list of i) included RCTs from eligible SRs, and ii) results from additional RCTs search. For duplicates or overlapping RCTs, we selected the most updated and comprehensive version for inclusion. To be included in NMA, RCTs should share a common comparator which serves as a bridge for indirect comparison between various peri-discharge complex interventions.

We used a pre-designed data extraction form to collect the following: year of publication, country, follow-up period of the study, interventions, comparators, number of patients analyzed and randomized in each group, patient age range, gender, and quantitative results of all pre-specified outcomes. When outcomes were reported in graphic forms, we used the GetData Graph Digitizer 2·26 software[1] to digitize and extract the required data.

References:

[1]. Fedorov S. GetData graph digitizer. *available at www getdata-graph-digitizer com* 2008.

Appendix 3 Definition of Coding Framework for simple components of peri-discharge complex interventions for reducing hospital readmissions

Simple Components	Codes	Definitions of coding an experimental intervention under the corresponding component
1. Discharge Planning	DP	Designing and implementing plans to get the patient ready for discharge
2. Case Management	CA	Coordinating care logistics and/or resources not specifically focused on enhancing patients' self-management capacity
3. Telephone Follow up	TE	Provider-initiated telephone or videophone communication after discharge with patients and / or the caregivers
4. Telemonitoring	TM	Applying mobile or internet technology for collecting objective measurements of patients' health status, with or without associated subjective assessment
5. Patient Education	PE	Education related to diagnosis or treatment targeted to the patient. It should not be focusing on enhancing self-management
6. Self-Management	SM	Patient-directed education or coaching aiming to empower patient's ability to manage his or her own care needs.
7. Medication Intervention	MI	Medication reconciliation or special education aimed at improving medication understanding or adherence; often but not necessarily conducted by a pharmacist.
8. Home Visits	HV	Physical visitation by intervention provider to patient's place of residence
9. Follow-Up Scheduled	FS	Scheduling of a follow-up visit prior to discharge
10. Patient Centered Discharge Instructions	PI	Improving the format or usability of discharge materials to make them more accessible or relevant compared to control
11. Provider Continuity	PC	Increased provider presence on both sides of the hospital to home transition. This may include involvement of GPs in inpatient care, or strategic follow up with inpatient provider after discharge, or coordination by a "bridging" healthcare provider

12. Timely Follow-up	TI	Post-discharge follow-up visit or communication with patient
13. Timely Primary Care Provider Communication	CM	Engagement with primary care providers in communication about patient status
14. Patient Hotline	PH	Presence of an telephone line for patient-initiated communication
15. Rehab Intervention	RI	Patient-directed rehabilitation efforts that are not entirely diagnosis-specific but aimed at improving functional statu
16. Streamlining	SL	A general streamlining of services provided, often with dedicated assignment of responsibility
17. Making Requisite	MR	Increasing the use or quality of services currently available but underutilized
18. Psychosocial services	PS	Psychosocial services are those services which aim to address the ongoing psychological and social needs of individuals. Within the health system services are generally provided to individuals with a disease or disorder, and/or their partners, families or caregivers.
19. Community services	CS	Involving multi-sector partnerships, include community members (e.g., lay health workers) as part of the intervention, and/or involve the delivery of services in community settings (e.g., schools, homes).
20. Other	-	Special situations unique to the intervention (caregiver education, peer mentoring, etc). Additional categories will be created if many interventions cannot be coded using the categories listed above

References of Appendix 3:

1. HansenLO,YoungRS,HinamiK,LeungA,WilliamsMV.Interventionstoreduce30-day rehospitalization: a systematic review. Annals of internal medicine. 2011;155(8):520-8.
2. Castillo E G, Ijadi-Maghsoodi R, Shadravan S, et al. Community Interventions to Promote Mental Health and Social Equity[J]. Current psychiatry reports, 2019, 21(5): 35.

Appendix 4 Flowchart of literature selection on additional search of randomized controlled trials (RCTs) of complex interventions for reducing 30-day readmissions among COPD patients

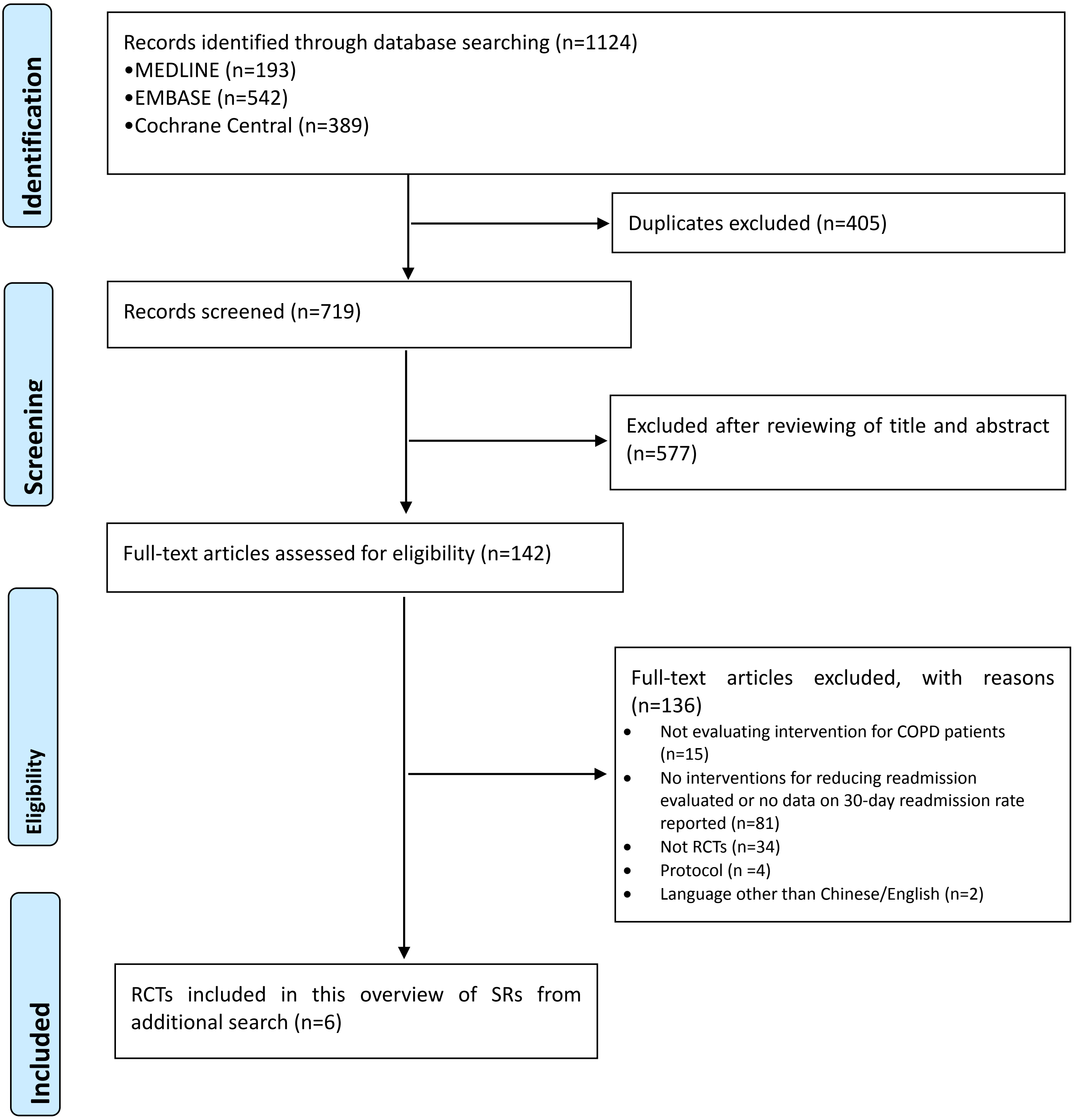

Keys: SRs: systematic reviews; RCTs: randomized controlled trials.

Appendix 5 Lists of included systematic reviews (SRs) and randomized controlled trials (RCTs)

Appendix 5a List of included SRs

1. Echevarria C, Brewin K, Horobin H, Bryant A, Corbett S, Steer J, et al. Early Supported Discharge/Hospital At Home For Acute Exacerbation of Chronic Obstructive Pulmonary Disease: A Review and Meta-Analysis. COPD. 2016;13(4):523-33.
2. Harrison SL, Janaudis-Ferreira T, Brooks D, Desveaux L, Goldstein RS. Self-management following an acute exacerbation of COPD: A systematic review. Chest. 2015;147(3):646-61.
3. Jeppesen E, Brurberg KG, Vist GE, Wedzicha JA, Wright JJ, Greenstone M, et al. Hospital at home for acute exacerbations of chronic obstructive pulmonary disease. The Cochrane database of systematic reviews. 2012(5):CD003573.
4. Majothi S, Jolly K, Heneghan NR, Price MJ, Riley RD, Turner AM, et al. Supported self-management for patients with COPD who have recently been discharged from hospital: a systematic review and meta-analysis. International journal of chronic obstructive pulmonary disease. 2015;10:853-67.
5. Ospina MB, Mrklas K, Deuchar L, Rowe BH, Leigh R, Bhutani M, et al. A systematic review of the effectiveness of discharge care bundles for patients with COPD. Thorax. 2017;72(1):31-9.
6. Pedersen PU, Ersgard KB, Soerensen TB, Larsen P. Effectiveness of structured planned post discharge support to patients with chronic obstructive pulmonary disease for reducing readmission rates: A systematic review. JBI Database of Systematic Reviews and Implementation Reports. 2017;15(8):2060-86.
7. Puhan MA, Gimeno-Santos E, Cates CJ, Troosters T. Pulmonary rehabilitation following exacerbations of chronic obstructive pulmonary disease. The Cochrane database of systematic reviews. 2016;12:CD005305.
8. Ryrso CK, Godtfredsen NS, Kofod LM, Lavesen M, Mogensen L, Tobberup R, et al. Lower mortality after early supervised pulmonary rehabilitation following COPD-exacerbations: a systematic review and meta-analysis. BMC pulmonary medicine. 2018;18(1):154.
9. Yang F, Xiong Z-F, Yang C, Li L, Qiao G, Wang Y, et al. Continuity of Care to Prevent Readmissions for Patients with Chronic Obstructive Pulmonary Disease: A Systematic Review and Meta-Analysis. COPD. 2017;14(2):251-61.

Appendix 5b List of included RCTs

1. Benzo R, Vickers K, Novotny PJ, Tucker S, Hoult J, Neuenfeldt P, et al. Health Coaching and Chronic Obstructive Pulmonary Disease Rehospitalization. A Randomized Study. American journal of respiratory and critical care medicine. 2016;194(6):672- 80.
2. Cotton MM, Bucknall CE, Dagg KD, Johnson MK, MacGregor G, Stewart C, et al. Early discharge for patients with exacerbations of chronic obstructive pulmonary disease: a randomized controlled trial. Thorax. 2000;55(11):902- 6.
3. Eaton T, Young P, Fergusson W, Moodie L, Zeng I, O'Kane F, et al. Does early pulmonary rehabilitation reduce acute health-care utilization in COPD patients admitted with an exacerbation? A randomized controlled study. Respirology (Carlton, Vic). 2009;14(2):230-8.
4. Hornikx M, Demeyer H, Camillo CA, Janssens W, Troosters T. The effects of a physical activity counseling program after an exacerbation in patients with Chronic Obstructive Pulmonary Disease: a randomized controlled pilot study. BMC pulmonary medicine. 2015;15:136.

5. Jakobsen AS, Laursen LC, Rydahl-Hansen S, Østergaard B, Gerds TA, Emme C, et al. Home-based telehealth hospitalization for exacerbation of chronic obstructive pulmonary disease: findings from "the virtual hospital" trial. Telemedicine journal and e-health. 2015;21(5):364- 73.
6. Jennings JH, Thavarajah K, Mendez MP, Eichenhorn M, Kvale P, Yessayan L. Predischarge bundle for patients with acute exacerbations of COPD to reduce readmissions and ED visits: a randomized controlled trial. Chest. 2015;147(5):1227-34.
7. Johnson-Warrington V, Rees K, Gelder C, Morgan MD, Singh SJ. Can a supported self-management program for COPD upon hospital discharge reduce readmissions? A randomized controlled trial. International journal of chronic obstructive pulmonary disease. 2016;11:1161- 9.
8. Kwok T, Lum CM, Chan HS, Ma HM, Lee D, Woo J. A randomized, controlled trial of an intensive community nurse-supported discharge program in preventing hospital readmissions of older patients with chronic lung disease. J Am Geriatr Soc. 2004;52(8):1240-6.
9. Lainscak M, Kadivec S, Kosnik M, Benedik B, Bratkovic M, Jakhel T, et al. Discharge coordinator intervention prevents hospitalizations in patients with COPD: a randomized controlled trial. J Am Med Dir Assoc. 2013;14(6):450 e1-6.
10. Lavesen M, Ladelund S, Frederiksen AJ, Lindhardt B, Overgaard D. Nurse-initiated telephone follow-up on patients with chronic obstructive pulmonary disease improves patient empowerment, but cannot prevent readmissions. Danish medical journal. 2016;63(10).
11. Wong KW, Wong FK, Chan MF. Effects of nurse-initiated telephone follow-up on self-efficacy among patients with chronic obstructive pulmonary disease. J Adv Nurs. 2005;49(2):210-22.

Appendix 6 Methodological quality of included systematic reviews (SRs) (n=9)

Items in AMSTAR 2	SRs (First author, year of publication)								
	Echevarria 2016	Harrison 2015	Jeppesen 2012	Majothi 2015	Ospina 2017	Pedersen 2017	Puhan 2016	Ryrso 2018	Yang 2017
1. Did the research questions and inclusion criteria for the review include the components of PICO?	PY	PY	Y	PY	PY	PY	Y	Y	PY
2. Did the report of the review contain an explicit statement that the review methods were established prior to the conduct of the review and did the report justify any significant deviations from the protocol?	PY	PY	Y	Y	Y	Y	Y	Y	N
3. Did the review authors explain their selection of the study designs for inclusion in the review?	N	N	N	N	N	N	N	N	N
4. Did the review authors use a comprehensive literature search strategy?	Y	PY	Y	PY	Y	Y	Y	Y	PY
5. Did the review authors perform study selection in duplicate?	Y	Y	Y	Y	Y	Y	Y	Y	Y
6. Did the review authors perform data extraction in duplicate?	N	Y	Y	Y	Y	Y	Y	Y	Y
7. Did the review authors provide a list of excluded studies and justify the exclusions?	Y	Y	Y	Y	Y	Y	Y	N	N
8. Did the review authors describe the included studies in adequate detail?	Y	Y	Y	Y	Y	Y	Y	Y	Y
9. Did the review authors use a satisfactory technique for	Y	Y	Y	Y	Y	Y	Y	Y	Y

Items in AMSTAR 2	SRs (First author, year of publication)								
	Echevarria 2016	Harrison 2015	Jeppesen 2012	Majothi 2015	Ospina 2017	Pedersen 2017	Puhan 2016	Ryrso 2018	Yang 2017
assessing the risk of bias (RoB) in individual studies that were included in the review?									
10. Did the review authors report on the sources of funding for the studies included in the review?	N	N	N	N	N	N	Y	N	N
11. If meta-analysis was performed did the review authors use appropriate methods for statistical combination of results?	Y	Y	Y	Y	Y	Y	Y	Y	Y
12. If meta-analysis was performed, did the review authors assess the potential impact of RoB in individual studies on the results of the meta-analysis or other evidence synthesis?	Y	N	Y	N	Y	N	Y	N	Y
13. Did the review authors account for RoB in individual studies when interpreting / discussing the results of the review?	Y	N	Y	Y	Y	Y	Y	Y	Y
14. Did the review authors provide a satisfactory explanation for, and discussion of, any heterogeneity observed in the results of the review?	Y	Y	Y	Y	N	Y	Y	Y	N
15. If they performed quantitative synthesis did the review authors carry out an adequate investigation of publication bias (small study bias) and discuss its likely	Y	N	N	N	N	N	N	N	Y

Items in AMSTAR 2	SRs (First author, year of publication)								
	Echevarria 2016	Harrison 2015	Jeppesen 2012	Majothi 2015	Ospina 2017	Pedersen 2017	Puhan 2016	Ryrso 2018	Yang 2017
impact on the results of the review?									
16. Did the review authors report any potential sources of conflict of interest, including any funding they received for conducting the review?	Y	Y	Y	Y	Y	Y	Y	Y	Y
Overall*	Moderate	Low	Moderate	Moderate	Moderate	Moderate	Moderate	Low	Critically low

Key: Y: Yes. PY: Partially yes. N: No. PICO: participants, interventions, comparisons, outcomes. RoB: risk of bias.

* Critical domains of AMSTAR 2 include item 2, item 4, item 7, item 9, item 11, item 13, item 15. For item 15, if the missing of test for publication bias is because of less than 10 individual studies, we will not consider it as a critical flaw.

Appendix 7a Risk of Bias among 11 included RCTs

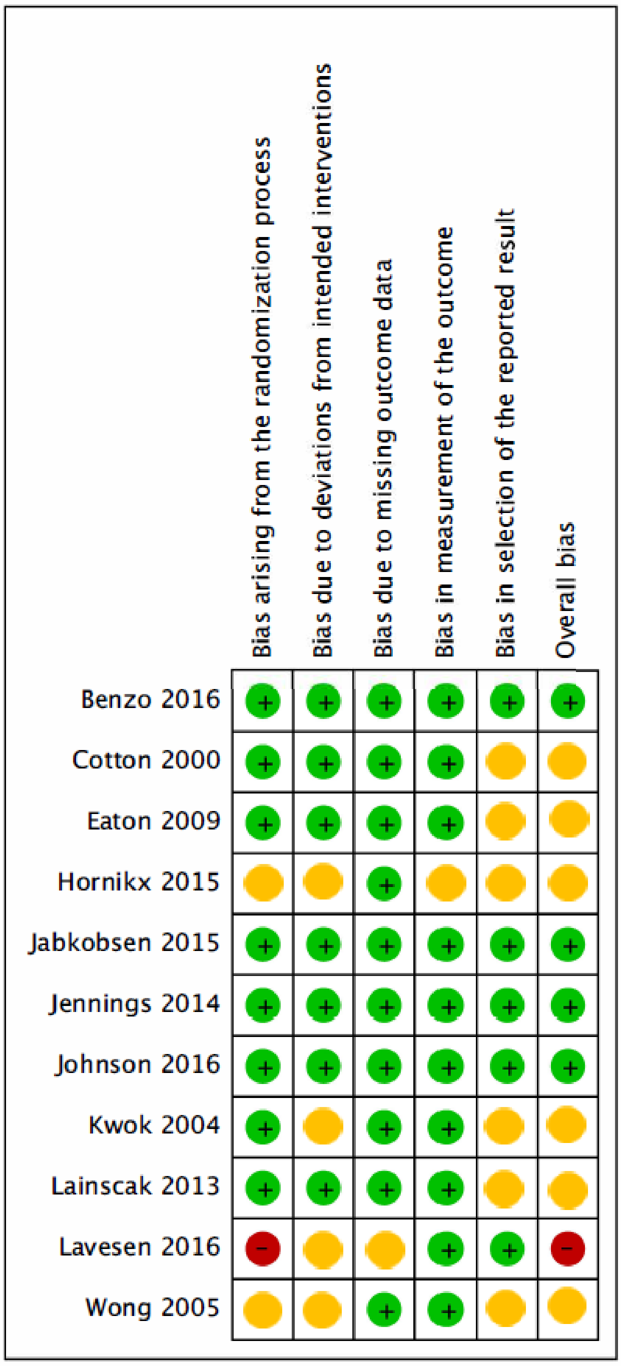

Notes: Green: low risk of bias; Yellow: some concerns; Red: high risk of bias.

Appendix 7b Detail results of each domain of risk of bias assessment using Risk of bias 2 for 11 RCTs

Domains	Signalling questions	Response of RCTs (n=11)										
		Benzo 2016	Cotton 2000	Eaton 2009	Hornikx 2015	Jabkobsen 2015	Jennings 2015	Johnson 2016	Kwok 2004	Lainscak 2013	Lavesen 2016	Wong 2005
Bias arising from the randomization process	1.1 Was the allocation sequence random?	Y	Y	Y	NI	Y	Y	Y	Y	Y	N	NI
	1.2 Was the allocation sequence concealed until participants were recruited and assigned to interventions?	NI	Y	Y	NI	Y	NI	Y	Y	Y	N	NI
	1.3 Did baseline differences between intervention groups suggest a problem with the randomization process?	N	N	N	N	N	N	N	N	N	N	N
	RoB judgement	Low	Low	Low	Some	Low	Low	Low	Low	Low	High	Some
Bias due to deviations from intended interventions	2.1. Were participants aware of their assigned intervention during the trial?	NI	NI	N	NI	Y	NI	Y	NI	NI	NI	NI
	2.2. Were carers and trial personnel aware of participants' assigned intervention during the trial?	N	NI	N	NI	Y	NI	Y	Y	NI	Y	N
	2.3. If Y/PY/NI to 2.1 or 2.2: Were there deviations from the intended intervention beyond what would be expected in usual practice?	N	N	NA	N	N	N	N	NI	N	N	NI
	2.4 If Y/PY to 2.3: Were these deviations likely to have affected the outcome?	NA	NA	NA	NA	NA	NA	NA	NA	NA	NA	NA
	2.5. If Y/PY/NI to 2.4: Were these deviations from intended intervention balanced between groups?	NA	NA	NA	NA	NA	NA	NA	NA	NA	NA	NA
	2.6 Was an appropriate analysis used to estimate the	Y	Y	Y	N	Y	Y	Y	N	Y	N	Y

Domains	Signalling questions	Response of RCTs (n=11)										
		Benzo 2016	Cotton 2000	Eaton 2009	Hornikx 2015	Jabkobsen 2015	Jennings 2015	Johnson 2016	Kwok 2004	Lainscak 2013	Lavesen 2016	Wong 2005
	effect of assignment to intervention?											
	2.7 If N/PN/NI to 2.6: Was there potential for a substantial impact (on the result) of the failure to analyse participants in the group to which they were randomized?	NA	NA	NA	N	NA	NA	NA	N	NA	N	NA
	RoB judgement	Low	Low	Low	Some	Low	Low	Low	Some	Low	Some	Some
Bias due to missing outcome data	3.1 Were outcome data available for all, or nearly all, participants randomized?	Y	Y	Y	Y	Y	Y	Y	Y	Y	N	Y
	3.2 If N/PN/NI to 3.1: Is there evidence that the result was not biased by missing outcome data?	NA	NA	NA	NA	NA	NA	NA	NA	NA	NI	NA
	3.3 If N/PN to 3.2: Could missingness in the outcome depend on its true value?	NA	NA	NA	NA	NA	NA	NA	NA	NA	NI	NA
	3.4 If Y/PY/NI to 3.3: Is it likely that missingness in the outcome depended on its true value?	NA	NA	NA	NA	NA	NA	NA	NA	NA	N	NA
	RoB judgement	Low	Low	Low	Low	Low	Low	Low	Low	Low	Some	Low
Bias in measurement of the outcome	4.1 Was the method of measuring the outcome inappropriate?	N	N	N	NI	N	N	N	N	N	N	N
	4.2 Could measurement or ascertainment of the outcome have differed between intervention groups?	N	N	N	NI	N	N	N	N	N	N	N
	4.3 If N/PN/NI to 4.1 and 4.2: Were outcome assessors aware of the intervention received by study participants?	N	NI	N	NI	Y	NI	N	Y	NI	N	N

Domains	Signalling questions	Response of RCTs (n=11)										
		Benzo 2016	Cotton 2000	Eaton 2009	Hornikx 2015	Jabkobsen 2015	Jennings 2015	Johnson 2016	Kwok 2004	Lainscak 2013	Lavesen 2016	Wong 2005
	4.4 If Y/PY/NI to 4.3: Could assessment of the outcome have been influenced by knowledge of intervention received?	NA	N	NA	N	N	N	NA	N	N	N	NA
	4.5 If Y/PY/NI to 4.4: Is it likely that assessment of the outcome was influenced by knowledge of intervention received?	NA	NA	NA	NA	NA	NA	NA	NA	NA	NA	NA
	RoB judgement	Low	Low	Low	Some	Low	Low	Low	Low	Low	Low	Low
Bias in selection of the reported result	5.1 Were the data that produced this result analysed in accordance with a pre-specified analysis plan that was finalized before unblinded outcome data were available for analysis?	Y	NI	NI	Y	Y	Y	Y	NI	N	Y	NI
	5.2. ... multiple eligible outcome measurements (e.g. scales, definitions, time points) within the outcome domain?	N	N	NI	NI	N	N	N	NI	N	N	NI
	5.3 ... multiple eligible analyses of the data?	N	N	NI	NI	N	N	N	NI	N	N	NI
	RoB judgement	Low	Some	Some	Some	Low	Low	Low	Some	Some	Low	Some
Overall bias		Low	Some	Some	Some	Low	Low	Low	Some	Some	High	Some

Notes: Y: Yes; N: No; PY: Probably Yes; PN: Probably No; NI: no information; NA: not applicable. Low: Low risk of bias; Some: Some concerns; High: High risk of bias; RoB: risk of bias judgement.

Appendix 8a. Pairwise meta-analyses of 9 RCTs: peri-discharge complex interventions versus controls, outcome: 30-day all-cause readmissions

Note: Peri-discharge complex interventions and controls are described in Table 1 and 2.

Study or Subgroup	Complex interventions Events	Total	Controls Events	Total	Weight	Risk Ratio M-H, Random, 95% CI
Benzo 2016	5	108	12	107	5.0%	0.41 [0.15, 1.13]
Cotton 2000	6	41	6	49	4.6%	1.20 [0.42, 3.42]
Hornikx 2015	4	15	6	15	4.7%	0.67 [0.23, 1.89]
Jabkobsen 2015	8	29	6	28	6.0%	1.29 [0.51, 3.24]
Jennings 2014	18	93	18	79	15.2%	0.85 [0.48, 1.52]
Kwok 2004	33	77	29	80	33.9%	1.18 [0.80, 1.74]
Lainscak 2013	7	118	8	135	5.3%	1.00 [0.37, 2.68]
Lavesen 2016	25	119	22	94	20.1%	0.90 [0.54, 1.49]
Wong 2005	5	30	8	30	5.2%	0.63 [0.23, 1.69]
**Total (95% CI)**		**630**		**617**	**100.0%**	**0.95 [0.76, 1.19]**
Total events	111		115			

Heterogeneity: Tau² = 0.00; Chi² = 5.83, df = 8 (P = 0.67); I² = 0%
Test for overall effect: Z = 0.47 (P = 0.64)

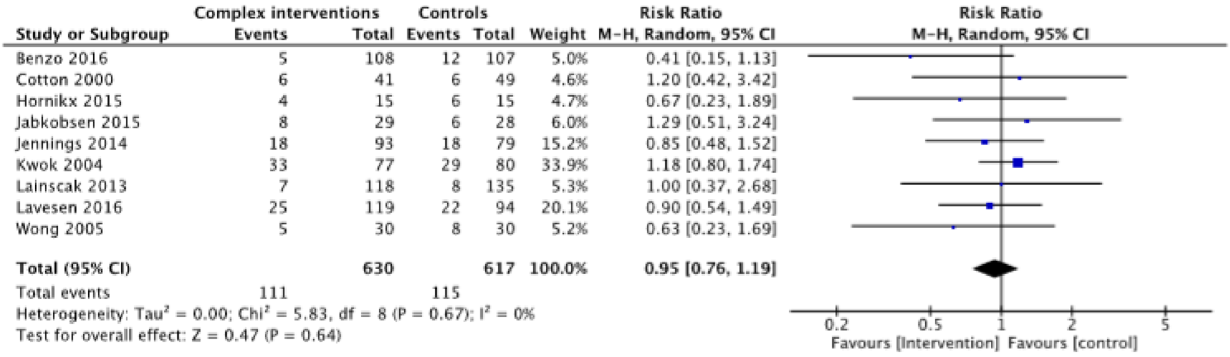

Appendix 8b. Pairwise meta-analyses of 4 RCTs: peri-discharge complex interventions versus controls, outcome: 30-day COPD-related readmissions

Note: Peri-discharge complex interventions and controls are described in Table 1 and 2.

Study or Subgroup	Complex interventions Events	Total	Controls Events	Total	Weight	Risk Ratio M-H, Random, 95% CI
Benzo 2016	2	108	10	107	17.5%	0.20 [0.04, 0.88]
Eaton 2009	3	47	4	50	18.7%	0.80 [0.19, 3.38]
Johnson 2016	5	39	10	39	40.8%	0.50 [0.19, 1.33]
Lainscak 2013	3	118	8	135	23.0%	0.43 [0.12, 1.58]
**Total (95% CI)**		**312**		**331**	**100.0%**	**0.45 [0.24, 0.84]**
Total events	13		32			

Heterogeneity: Tau² = 0.00; Chi² = 1.84, df = 3 (P = 0.61); I² = 0%
Test for overall effect: Z = 2.52 (P = 0.01)

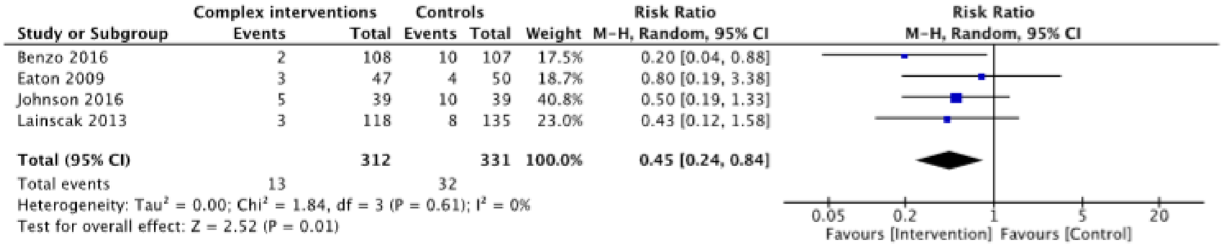

Appendix 8c. Pairwise meta-analyses among 5 RCTs: peri-discharge complex interventions versus controls, outcome: 3-month all-cause readmissions

Note: Peri-discharge complex interventions and controls are described in Table 1 and 2.

Study or Subgroup	Experimental Events	Total	Control Events	Total	Weight	Risk Ratio M-H, Random, 95% CI
Benzo 2016	15	108	27	107	20.2%	0.55 [0.31, 0.97]
Eaton 2009	11	47	16	50	15.3%	0.73 [0.38, 1.41]
Jabkobsen 2015	10	29	11	28	14.2%	0.88 [0.44, 1.73]
Johnson 2016	12	39	13	39	15.8%	0.92 [0.48, 1.76]
Lainscak 2013	25	118	39	135	34.5%	0.73 [0.47, 1.14]
**Total (95% CI)**		**341**		**359**	**100.0%**	**0.74 [0.57, 0.95]**
Total events	73		106			

Heterogeneity: Tau² = 0.00; Chi² = 1.73, df = 4 (P = 0.78); I² = 0%
Test for overall effect: Z = 2.34 (P = 0.02)

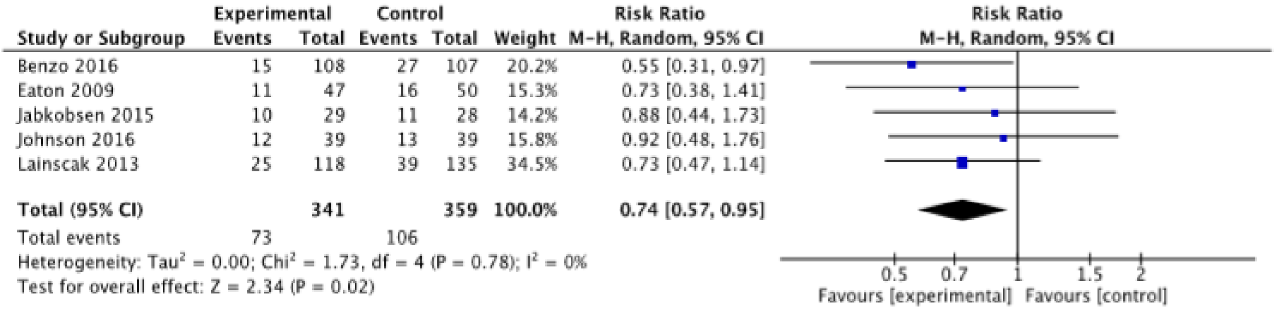

Appendix 8d. Pairwise meta-analyses among 4 RCTs: peri-discharge complex interventions versus controls, outcome: 6-month all-cause readmissions

Note: Peri-discharge complex interventions and controls are described in Table 1 and 2.

	Experimental		Control			Risk Ratio
Study or Subgroup	Events	Total	Events	Total	Weight	M-H, Random, 95% CI
Benzo 2016	28	108	40	107	23.1%	0.69 [0.46, 1.04]
Jabkobsen 2015	13	29	14	28	16.7%	0.90 [0.52, 1.55]
Kwok 2004	53	77	49	80	32.9%	1.12 [0.89, 1.41]
Lainscak 2013	37	118	60	135	27.2%	0.71 [0.51, 0.98]
**Total (95% CI)**		**332**		**350**	**100.0%**	**0.85 [0.64, 1.14]**
Total events	131		163			

Heterogeneity: $Tau^2 = 0.05$; $Chi^2 = 8.00$, df = 3 (P = 0.05); $I^2 = 63\%$
Test for overall effect: Z = 1.08 (P = 0.28)

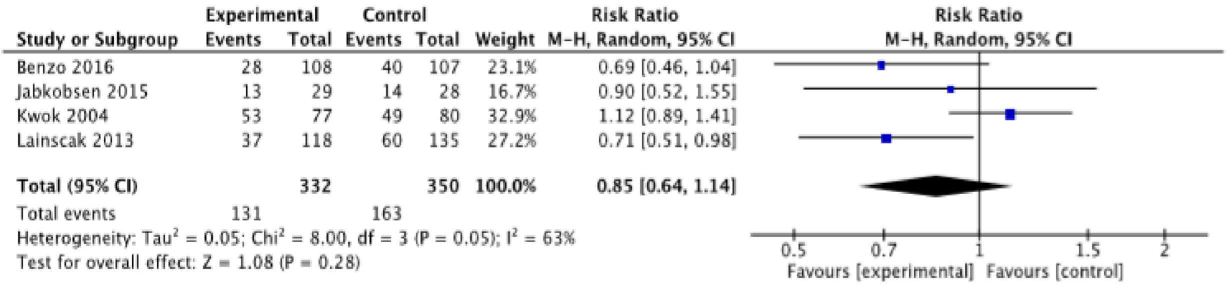

Appendix 9. Pairwise meta-analyses among 2 RCTs: peri-discharge complex interventions versus controls, outcome: 30-day mortality

Note: Peri-discharge complex interventions and controls are described in Table 1 and 2.

	Experimental		Control			Risk Ratio
Study or Subgroup	Events	Total	Events	Total	Weight	M-H, Random, 95% CI
Lainscak 2013	1	118	6	135	41.4%	0.19 [0.02, 1.56]
Lavesen 2016	2	119	3	94	58.6%	0.53 [0.09, 3.09]
**Total (95% CI)**		**237**		**229**	**100.0%**	**0.35 [0.09, 1.34]**
Total events	3		9			

Heterogeneity: $Tau^2 = 0.00$; $Chi^2 = 0.54$, df = 1 (P = 0.46); $I^2 = 0\%$
Test for overall effect: Z = 1.54 (P = 0.12)

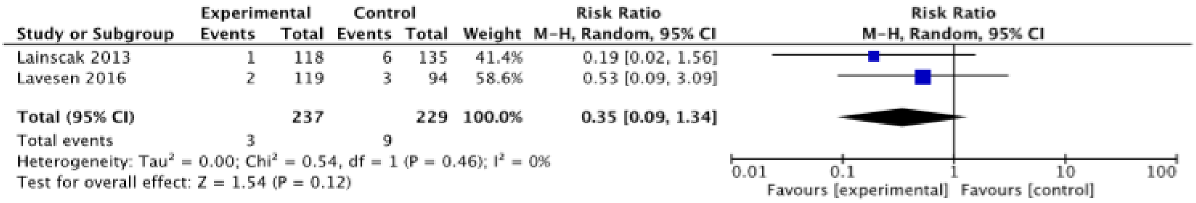

Appendix 10. Sensitivity analysis focusing on 3 RCTs with overall low risk of bias: peri-discharge complex interventions versus controls, outcome: 30-day all-cause readmissions

Note: Peri-discharge complex interventions and controls are described in Table 1 and 2.

	Complex intervention		Control			Risk Ratio
Study or Subgroup	Events	Total	Events	Total	Weight	M-H, Random, 95% CI
Benzo 2016	5	108	12	107	23.2%	0.41 [0.15, 1.13]
Jabkobsen 2015	8	29	6	28	26.7%	1.29 [0.51, 3.24]
Jennings 2014	18	93	18	79	50.0%	0.85 [0.48, 1.52]
**Total (95% CI)**		**230**		**214**	**100.0%**	**0.80 [0.47, 1.38]**
Total events	31		36			

Heterogeneity: $Tau^2 = 0.07$; $Chi^2 = 2.74$, df = 2 (P = 0.25); $I^2 = 27\%$
Test for overall effect: Z = 0.79 (P = 0.43)

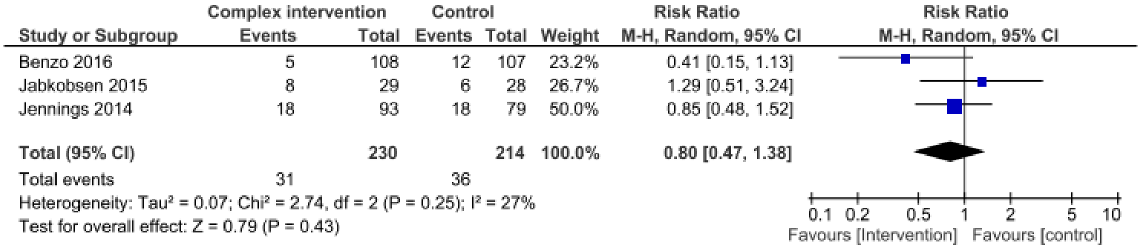

Appendix 11. Subgroup analysis based on different comparisons in the control group, outcome: 30-day all-cause readmissions

Note: Comparisons in the control group include rehabilitation education, and usual care. Usual care is defined as routine care provided by the hospital.

	Complex intervention		Control			Risk Ratio
Study or Subgroup	Events	Total	Events	Total	Weight	M-H, Random, 95% CI
**5.1.2 Complex intervention vs. Rehabilitation Education**						
Hornikx 2015	4	15	6	15	9.2%	0.67 [0.23, 1.89]
Lainscak 2013	7	118	8	135	10.4%	1.00 [0.37, 2.68]
**Subtotal (95% CI)**		**133**		**150**	**19.6%**	**0.83 [0.40, 1.69]**
Total events	11		14			
Heterogeneity: Tau² = 0.00; Chi² = 0.32, df = 1 (P = 0.57); I² = 0%						
Test for overall effect: Z = 0.52 (P = 0.60)						
**5.1.3 Complex interventions vs. usual care**						
Benzo 2016	5	108	12	107	9.9%	0.41 [0.15, 1.13]
Cotton 2000	6	41	6	49	9.1%	1.20 [0.42, 3.42]
Jabkobsen 2015	8	29	6	28	11.8%	1.29 [0.51, 3.24]
Lavesen 2016	25	119	22	94	39.4%	0.90 [0.54, 1.49]
Wong 2005	5	30	8	30	10.1%	0.63 [0.23, 1.69]
**Subtotal (95% CI)**		**327**		**308**	**80.4%**	**0.85 [0.60, 1.21]**
Total events	49		54			
Heterogeneity: Tau² = 0.00; Chi² = 3.58, df = 4 (P = 0.47); I² = 0%						
Test for overall effect: Z = 0.91 (P = 0.36)						
**Total (95% CI)**		**460**		**458**	**100.0%**	**0.84 [0.61, 1.16]**
Total events	60		68			
Heterogeneity: Tau² = 0.00; Chi² = 3.88, df = 6 (P = 0.69); I² = 0%						
Test for overall effect: Z = 1.04 (P = 0.30)						
Test for subgroup differences: Chi² = 0.00, df = 1 (P = 0.95), I² = 0%						

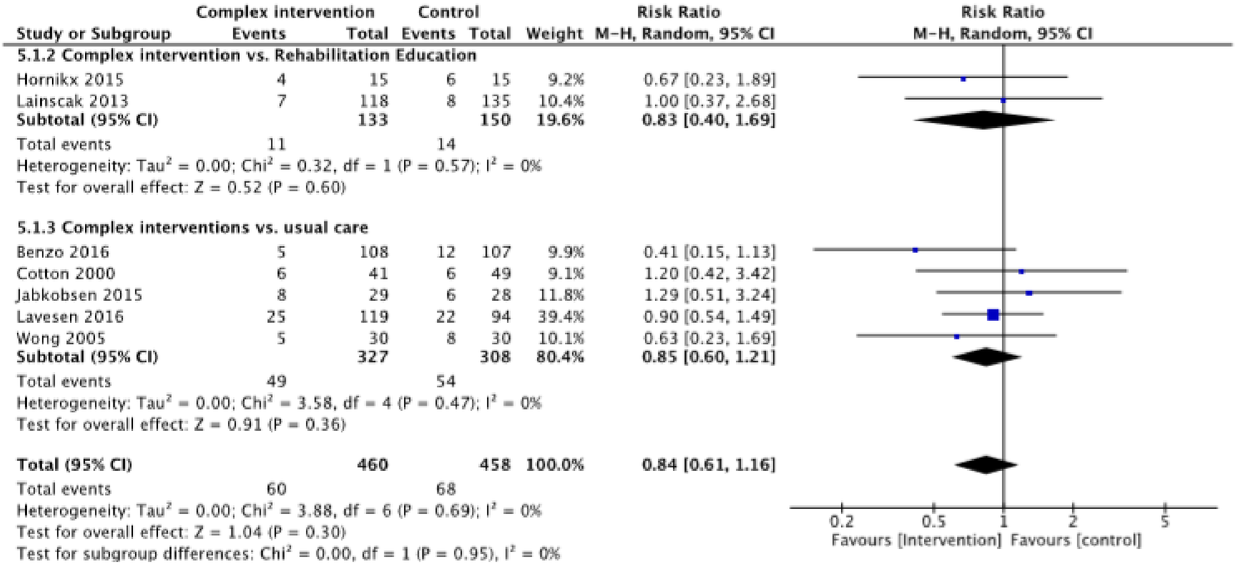

Appendix 12. Comparative effectiveness of 8 different peri-discharge complex interventions and usual care for reducing 30-day all-cause readmissions among COPD patients

Notes: Peri-discharge complex interventions and usual care are described in Table 1 and 2. The values in each cell represent odds ratio (OR, and 95% confidence interval) of the intervention at the top, compared to the comparator on the left. When OR<1, prefers the column intervention, indicating that the column intervention could be more effective than the row intervention on reducing 30-day all-cause readmissions; When OR>1, prefers the row intervention.

Usual care								
1.82(0.52,6.38)	Supported self-management program							
0.72(0.21,2.41)	0.39(0.07,2.26)	Home based telemedicine						
0.39(0.05,2.78)	0.21(0.02,2.21)	0.55(0.12,2.55)	Rehabilitation education					
2.40(0.59,9.82)	1.32(0.69,2.51)	3.35(0.52,21.55)	6.14 (0.55,68.85)	Follow up appointment				
1.06(0.60,1.89)	0.59(0.15,2.33)	1.49(0.39,5.70)	2.72(0.35,21.07)	0.44(0.10,2.03)	Early discharge intervention			
2.60(0.88,7.66)	1.43(0.27,7.50)	3.63(0.71,18.48)	6.66(0.71,62.63)	1.09(0.18,6.41)	2.45 (0.72,8.30)	Discharge rehabilitation		
0.32(0.03,3.30)	0.17 (0.01,2.49)	0.44(0.06,3.38)	0.81(0.23,2.92)	0.13(0.01,2.04)	0.30(0.03,3.33)	0.12(0.01,1.61)	Discharge education	
0.39(0.04,3.61)	0.21(0.02,2.76)	0.54(0.08,3.51)	1.00(0.35,2.84)	0.16(0.01,2.27)	0.37(0.04,3.65)	0.15(0.01,1.78)	1.23(0.59,2.57)	Discharge coordinator intervention

Appendix 13 Quality of evidence ratings on network meta-analysis effect for comparison of the 8 different peri-discharge complex interventions and usual care for reducing 30-day all-cause readmissions among COPD patients

Comparison	Number of studies*	Study limitation**	Inconsistency †	Indirectness	Imprecision	Publication bias	Network estimate: OR (95%CI)	QualityΨ
Discharge Coordinator Intervention vs. Discharge Education	1	No concerns	No concerns	No concerns	Major concernsΔΔ	N/A	0.81(0.39,1.69)	Low
Discharge Coordinator Intervention vs. Rehabilitation Education	1	Some concerns	No concerns	No concerns	Major concernsΔΔ	N/A	1.00(0.35,2.84)	Low
Discharge rehabilitation vs. Usual care	1	No concerns	No concerns	No concerns	Some concernsΔ	N/A	0.38(0.13,1.14)	Moderate
Early discharge intervention vs. Usual care	2	Major concerns	No concerns	No concerns	Major concernsΔΔ	N/A	0.94(0.53,1.67)	Low
Follow up Appointment vs. Supported self-management program	1	Some concerns	No concerns	No concerns	Some concernsΔ	N/A	0.76(0.40,1.45)	Moderate
Home based telemedicine vs. Rehabilitation Education	1	Some concerns	No concerns	No concerns	Major concernsΔΔ	N/A	0.55(0.12,2.55)	Low
Home based telemedicine vs. Usual care	1	No concerns	No concerns	No concerns	Major concernsΔΔ	N/A	1.39(0.41,4.76)	Low
Supported self-management program vs. Usual care	1	Some concerns	No concerns	No concerns	Major concernsΔΔ	N/A	0.55(0.16,1.92)	Low
Discharge Coordinator Intervention vs. Discharge rehabilitation	0	Some concerns	No concerns	No concerns	Major concernsΔΔ	N/A	6.67(0.56,100)	Low
Discharge Coordinator Intervention vs. Early discharge intervention	0	Some concerns	No concerns	No concerns	Major concernsΔΔ	N/A	2.70(0.27,25.0)	Low
Discharge Coordinator Intervention vs. Follow up Appointment	0	Some concerns	No concerns	No concerns	Major concernsΔΔ	N/A	6.25(0.44,100)	Low
Discharge Coordinator Intervention vs. Home based telemedicine	0	Some concerns	No concerns	No concerns	Major concernsΔΔ	N/A	1.85(0.28,12.5)	Low

Comparison	Number of studies*	Study limitation**	Inconsistency †	Indirectness	Imprecision	Publication bias	Network estimate: OR (95%CI)	QualityΨ
Discharge Coordinator Intervention vs. Supported self-management program	0	Some concerns	No concerns	No concerns	Major concernsΔΔ	N/A	4.76(0.36,50.0)	Low
Discharge Coordinator Intervention vs. Usual care	0	Some concerns	No concerns	No concerns	Major concernsΔΔ	N/A	2.56(0.28,25.0)	Low
Discharge Education vs. Discharge rehabilitation	0	No concerns	No concerns	No concerns	Major concernsΔΔ	N/A	8.33(0.62,100)	Low
Discharge Education vs. Early discharge intervention	0	Some concerns	No concerns	No concerns	Major concernsΔΔ	N/A	3.33(0.30,33.3)	Low
Discharge Education vs. Follow up Appointment	0	Some concerns	No concerns	No concerns	Major concernsΔΔ	N/A	7.69(0.49,100)	Low
Discharge Education vs. Home based telemedicine	0	Some concerns	No concerns	No concerns	Major concernsΔΔ	N/A	2.27(0.30,16.7)	Low
Discharge Education vs. Rehabilitation Education	0	Some concerns	No concerns	No concerns	Major concernsΔΔ	N/A	1.23(0.34,4.35)	Low
Discharge Education vs. Supported self-management program	0	Some concerns	No concerns	No concerns	Major concernsΔΔ	N/A	5.88(0.40,100)	Low
Discharge Education vs. Usual care	0	Some concerns	No concerns	No concerns	Major concernsΔΔ	N/A	3.13(0.30,33.3)	Low
Discharge rehabilitation vs. Early discharge intervention	0	Some concerns	No concerns	No concerns	Some concernsΔ	N/A	0.41(0.12,1.39)	Moderate
Discharge rehabilitation vs. Follow up Appointment	0	Some concerns	No concerns	No concerns	Major concernsΔΔ	N/A	0.92(0.16,5.56)	Low
Discharge rehabilitation vs. Home based telemedicine	0	No concerns	No concerns	No concerns	Some concernsΔ	N/A	0.28(0.05,1.41)	Moderate
Discharge rehabilitation vs. Rehabilitation Education	0	No concerns	No concerns	No concerns	Some concernsΔ	N/A	0.15(0.02,1.41)	Moderate
Discharge rehabilitation vs. Supported self-management program	0	Some concerns	No concerns	No concerns	Major concernsΔΔ	N/A	0.70(0.13,3.70)	Low
Early discharge intervention vs. Follow up Appointment	0	Some concerns	No concerns	No concerns	Major concernsΔΔ	N/A	2.27(0.49,10.0)	Low
Early discharge intervention vs. Home based telemedicine	0	Some concerns	No concerns	No concerns	Major concernsΔΔ	N/A	0.67(0.18,2.56)	Low

Comparison	Number of studies*	Study limitation**	Inconsistency †	Indirectness	Imprecision	Publication bias	Network estimate: OR (95%CI)	QualityΨ
Early discharge intervention vs. Rehabilitation Education	0	Some concerns	No concerns	No concerns	Major concernsΔΔ	N/A	0.37(0.05,2.86)	Low
Early discharge intervention vs. Supported self-management program	0	Some concerns	No concerns	No concerns	Major concernsΔΔ	N/A	1.69(0.43,6.67)	Low
Follow up Appointment vs. Home based telemedicine	0	Some concerns	No concerns	No concerns	Major concernsΔΔ	N/A	0.30(0.05,1.92)	Low
Follow up Appointment vs. Rehabilitation Education	0	Some concerns	No concerns	No concerns	Major concernsΔΔ	N/A	0.16(0.01,1.82)	Low
Follow up Appointment vs. Usual care	0	Some concerns	No concerns	No concerns	Major concernsΔΔ	N/A	0.42(0.10,1.69)	Low
Home based telemedicine vs. Supported self-management program	0	Some concerns	No concerns	No concerns	Major concernsΔΔ	N/A	2.56(0.44,14.3)	Low
Rehabilitation Education vs. Supported self-management program	0	Some concerns	No concerns	No concerns	Major concernsΔΔ	N/A	4.76(0.45,50.0)	Low
Rehabilitation Education vs. Usual care	0	Some concerns	No concerns	No concerns	Major concernsΔΔ	N/A	2.56(0.36,20.0)	Low

Notes: OR: odds ratio; CI: confidence interval.
*: Number of studies equals to 0, indicating that there were no direct comparisons within the included studies.
**: Study limitation is calculated by weighting risk of bias based on the contribution of direct estimates to the network.
†: We consider inconsistency by statistical inconsistency. In this network, no significant statistical inconsistency is detected by SIDE approach. (Appendix 13).
Δ: Serious imprecision caused by the 95% CI overlaps the OR of 1.0 but includes important benefit or important harm (OR estimates below 0.667 and above1.5 are considered clinically important).
ΔΔ: Very serious imprecision caused by the 95% CI overlaps the OR of 1.0 but includes both important benefit and important harm (OR estimates below 0.667 and above 1.5 are considered clinically important).
Ψ: The quality of evidence starts from high quality and is downgraded by one level for each domain with some concerns, and by two levels for each domain with major concerns. As the domains of study limitation, inconsistency, and imprecision are interconnected, we only downgrade the overall level of quality for the most serious concerns in the domains to avoid downgrading more than once for related concerns.
N/A: Not applicable for publication bias because of less than 10 individual studies.

Appendix 14. Comparative effectiveness of 8 different peri-discharge complex interventions and usual care: surface under the cumulative ranking curves (SUCRA) for reducing 30-day all-cause readmissions among COPD patients

Note: Peri-discharge complex interventions and usual care are described in Table 1 and 2.The *x*-axis represents the possible rank of each intervention (from the first best rank to the worst according to effectiveness on reducing 30-day all-cause readmissions). The *y*-axis indicates the cumulative probability for each intervention to be the best intervention, the second-best intervention, the third best intervention, and so on. In this graphical approach, rankings are presented through examining the area under the curve. The bigger the area under the curve, the higher the likelihood that an intervention is in the top rank or one of the top ranks.

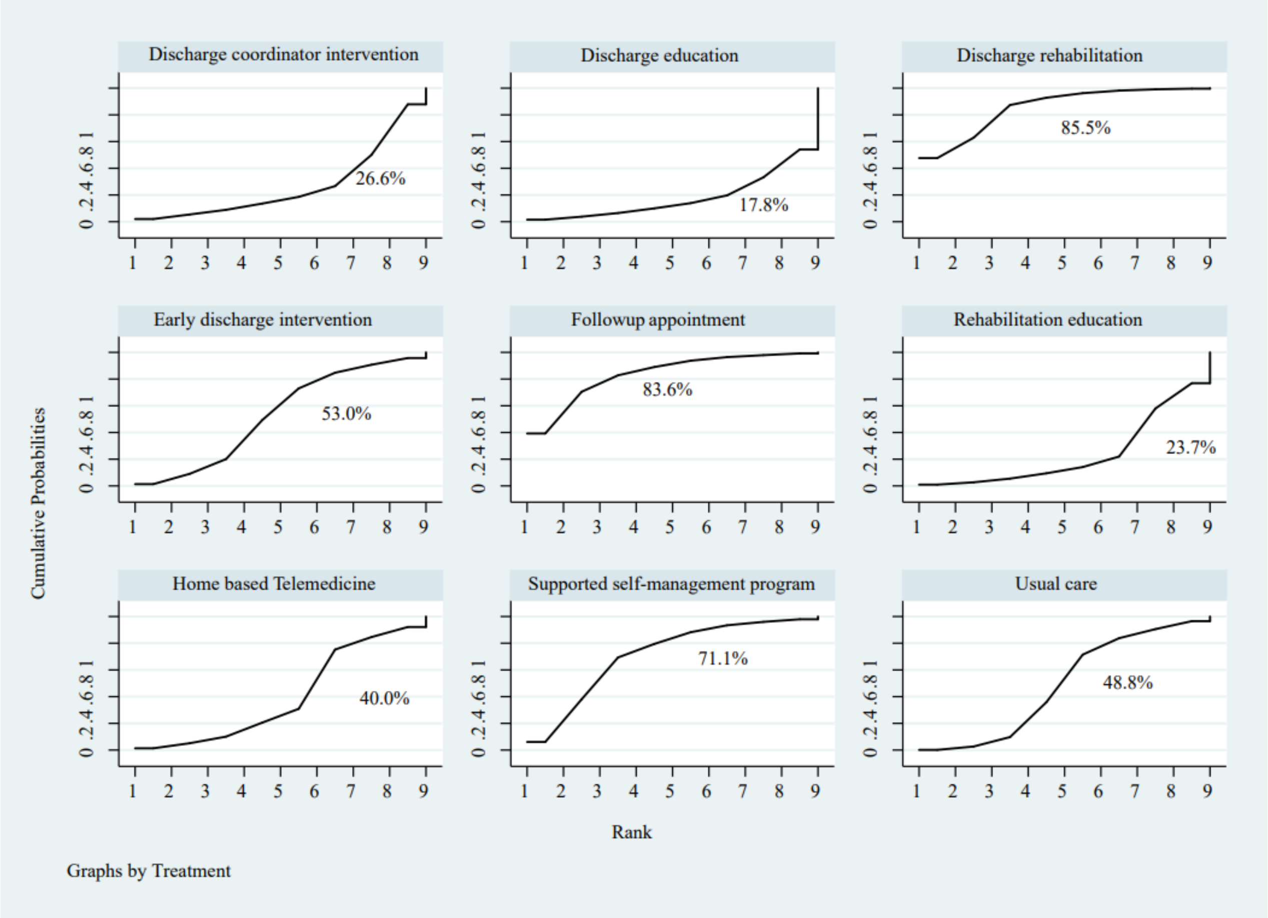

Appendix 15 Final classification of the complex interventions based on network meta-analysis on reducing 30-day all-cause readmissions

Quality of the evidence, and classification of the intervention	Peri-discharge complex intervention	Peri-discharge complex intervention versus usual care (OR (95% CI))	SUCRA curve	SUCRA Rank
**High certainty category (moderate or high quality of evidence)**				
Group 2: interventions which are superior to at least one intervention in Group 1	-	-	-	-
Group 1: interventions which are not differ from usual care	Discharge rehabilitation	0.38(0.13,1.14)	85.5%	1
**Low certainty category (low or very low quality of evidence)**				
Group 2: interventions which might be superior to at least one intervention in Group 1	-	-	-	-
Group 1: interventions which might not be different from usual care	Follow up appointment	0.42(0.10,1.69)	83.6%	2
	Supported self-management program	0.55(0.16,1.92)	71.1%	3
	Early discharge intervention	0.94(0.53,1.67)	53.0%	4
	Home based telemedicine	1.39(0.41,4.76)	40.0%	5
	Discharge coordinator intervention	2.56(0.28,25.0)	26.6%	6
	Rehabilitation education	2.56(0.36,20.0)	23.7%	7
	Discharge education	3.13(0.30,33.3)	17.8%	8

Notes: OR: odds ratio; CI: confidence interval. SUCRA: Surface under the cumulative ranking curve.

Appendix 16 Results based on the Separating Indirect from Direct Evidence (SIDE) approach to evaluating local inconsistency in NMA for reducing 30-day all-cause readmission among COPD patients

Comparison	Direct		Indirect		Inconsistency factor		
	Estimate	Standard error	Estimate	Standard error	Estimate	Standard error	P
Discharge Coordinator Intervention vs. Rehabilitation Education	0.00	0.53	1.01	92.16	-1.01	92.17	0.99
Discharge rehabilitation vs. Usual care	0.96	0.55	-1.70	744.95	2.66	744.95	1.00
Early discharge intervention vs. Usual care	0.06	0.29	-1.89	369.08	1.95	369.08	1.00
Follow up Appointment vs. Supported self-management program	0.28	0.33	-3.08	407.52	3.36	407.52	0.99
Rehabilitation Education vs. Home based telemedicine	-0.61	0.79	0.54	99.31	-1.15	99.31	0.99
Home based telemedicine vs. Usual care	-0.33	0.62	0.96	110.98	-1.29	110.98	0.99
Supported self-management program vs. Usual care	0.60	0.64	-1.19	188.29	1.79	188.29	0.99

Notes: P values less than 0.05 suggest statistically significant inconsistency.
